# VAMP7 regulates constitutive membrane incorporation of the cold-activated channel TRPM8

**DOI:** 10.1038/ncomms10489

**Published:** 2016-02-04

**Authors:** Debapriya Ghosh, Silvia Pinto, Lydia Danglot, Ine Vandewauw, Andrei Segal, Nele Van Ranst, Melissa Benoit, Annelies Janssens, Rudi Vennekens, Pieter Vanden Berghe, Thierry Galli, Joris Vriens, Thomas Voets

**Affiliations:** 1 Laboratory of Ion Channel Research and TRP Channel Research Platform Leuven (TRPLe), Department of Cellular and Molecular Medicine, University of Leuven, Herestraat 49 box 802, B-3000 Leuven, Belgium; 2 Institut Jacques Monod, CNRS UMR 7592, University of Paris Diderot, F-75013 Paris, France; 3 INSERM ERL U950, Membrane Traffic in Health & disease Group, F-75013 Paris, France; 4 Laboratory for Enteric NeuroScience, Department of Clinical and Experimental Medicine, University of Leuven, B-3000 Leuven, Belgium; 5 Translational Research Centre for Gastrointestinal Disorders, KU Leuven, Herestraat 49 box 701, B-3000 Leuven, Belgium; 6 Laboratory of Experimental Gynaecology, Department of Development and Regeneration, University of Leuven, Herestraat 49 box 611, B-3000 Leuven, Belgium

## Abstract

The cation channel TRPM8 plays a central role in the somatosensory system, as a key sensor of innocuously cold temperatures and cooling agents. Although increased functional expression of TRPM8 has been implicated in various forms of pathological cold hypersensitivity, little is known about the cellular and molecular mechanisms that determine TRPM8 abundance at the plasma membrane. Here we demonstrate constitutive transport of TRPM8 towards the plasma membrane in atypical, non-acidic transport vesicles that contain lysosomal-associated membrane protein 1 (LAMP1), and provide evidence that vesicle-associated membrane protein 7 (VAMP7) mediates fusion of these vesicles with the plasma membrane. In line herewith, VAMP7-deficient mice exhibit reduced functional expression of TRPM8 in sensory neurons and concomitant deficits in cold avoidance and icilin-induced cold hypersensitivity. Our results uncover a cellular pathway that controls functional plasma membrane incorporation of a temperature-sensitive TRP channel, and thus regulates thermosensitivity *in vivo*.

## Abstract (web summary)

The temperature-sensitive TRPM8 channel is essential for cold sensing and has been linked to pathological cold hypersensitivity. Here, the authors find TRPM8 insertion in the cell membrane is mediated by VAMP7 following atypical LAMP1-containing vesicle transport, and that loss of VAMP7 leads to reduced cold avoidance *in vivo*.

## Graphical abstract

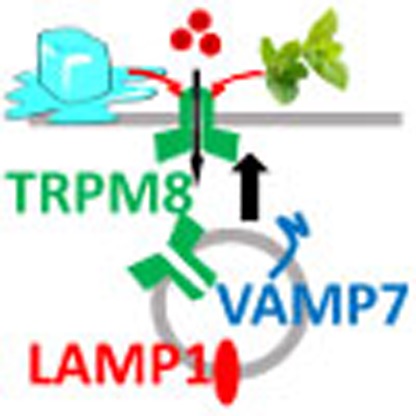

The transient receptor potential (TRP) cation channel TRPM8 plays a central role in thermosensation^1^. TRPM8 is expressed in a subset of sensory neurons, where it acts as a direct sensor of cold stimuli and cooling agents such as menthol or icilin^2^,^3^,^4^,^5^,^6^. As a consequence, genetic ablation or pharmacological inhibition of TRPM8 leads to specific deficits in the avoidance of innocuously cold temperatures^2^,^3^,^4^,^7^,^8^. Increased functional expression of TRPM8 contributes to pathological cold hypersensitivity and cold allodynia in various animal models of neuropathic and inflammatory pain^3^,^8^,^9^. Oppositely, activation of TRPM8 was shown to mediate the analgesic effect of cooling and menthol in acute or inflammatory pain^4^,^10^. As such, directed manipulation of TRPM8 activity may have therapeutic potential in the treatment of cold dysesthesias in patients.

In recent years, important advances have been made in understanding the gating of TRPM8, including mechanisms that control the changes in open probability of TRPM8 in response to cold or cooling agents^11^,^12^,^13^,^14^,^15^, and its regulation by cellular signalling pathways^16^,^17^,^18^. In comparison, knowledge about the mechanisms that determine the abundance of TRPM8 at the plasma membrane is sparse, as is the case for most other TRP channels. Nevertheless, modulation of the number of active TRP channels at the plasma membrane represents an important regulatory mechanism under normal and pathophysiological conditions^19^,^20^,^21^. Moreover, recent research indicates that acute stimulation of TRPA1 (ref. 22) and TRPM8 (ref. 23) not only increases the open probability of channels at the plasma membrane, but can also stimulate incorporation of new channels through exocytosis. However, the cellular structures and molecular determinants that govern the transport of these channels to the plasma membrane are largely unknown.

SNAREs (soluble *N*-ethylmaleimide-sensitive factor attachment protein receptors) are transmembrane proteins essential for the fusion of lipid bilayers^24^. VAMP7 (also known as tetanus neurotoxin-insensitive VAMP or TI-VAMP) participates in exocytosis of Golgi-derived vesicles^25^, retromer-derived endosomal vesicles^26^ and lysosomal-related organelles^27^. VAMP7-deficient mice exhibit a lowered core body temperature, suggesting a potential role for VAMP7 in thermosensation and/or thermoregulation^28^, but a mechanistic link between VAMP7 function and thermosensation/thermoregulation remains elusive.

Here we have combined Total Internal Reflection Fluorescence (TIRF) imaging with functional and behavioural assays to identify mechanisms that underlie transport of TRPM8 to the plasma membrane. We demonstrate transport of TRPM8 to the plasma membrane via an atypical vesicular compartment positive for LAMP1, and provide evidence implicating VAMP7 in the fusion of these vesicles with the plasma membrane. Finally, we show that VAMP7-deficient mice exhibit reduced TRPM8 activity in sensory neurons, associated with impaired cold avoidance behaviour.

## Results

### Rapid microtubular transport of TRPM8

**Figure 1:**
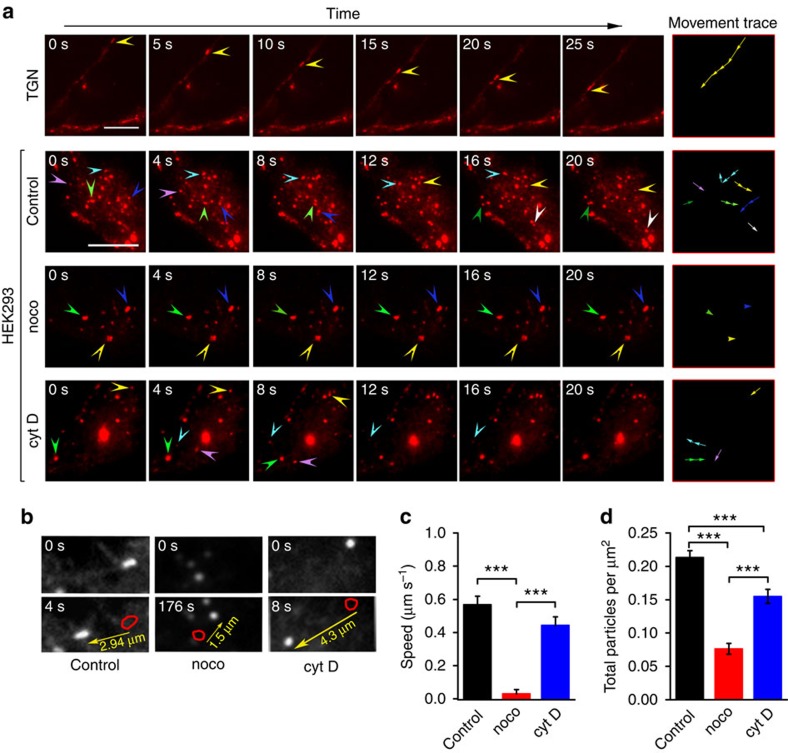
Rapid vesicular transport of TRPM8 via microtubules. (**a**) TIRF images at consecutive intervals showing the movement of TRPM8–mCherry in TGN, control HEK293 cells and HEK293 cells treated with nocodazole (30 μM) and cytochalasin D (2 μM). The arrowheads point at moving TRPM8-positive structures. The rightmost column depicts the movement trace of the structures highlighted by the arrowheads. Scale bar, 10 μm. (**b**) TIRF images for control, nocodazole-treated and cytochalasin D-treated HEK293 cells illustrating the movement of TRPM8-positive structures in the indicated time interval, allowing calculation of the movement speed. The arrow indicates the direction and distance of movement. (**c**) Average speed of movement of the TRPM8-positive structures in control (*n*=26) cells and cells treated with nocodazole (*n*=20) and cytochalasin D (*n*=20) following the procedure depicted in **b**. (**d**) Quantification of the density of TRPM8-positive structures in control cells and cells treated with nocodazole and cytochalasin D. Significance was determined by 1-way ANOVA with Tukey's *post hoc* test; ***P<0.001.

To analyse the near-membrane intracellular transport of TRPM8, we transfected HEK293 cells and mouse trigeminal ganglion neurons (TGNs) with a plasmid encoding TRPM8 with mCherry fused to its C terminus (TRPM8–mCherry), and used TIRF microscopy to monitor the movement of fluorescent structures in close proximity of the plasma membrane. In line with earlier work^29^, TIRF imaging revealed a population of highly dynamic vesicular and tubular structures, which composed the bulk of the fluorescent signal (Fig. 1a). Tracking of XY-position of these TRPM8 structures yielded an average speed of 0.58±0.08 μm s^−1^ (Fig. 1a–c).

To investigate the nature of the cytoskeletal tracks via which the dynamic, TRPM8-containing structures move, their number and speed was compared between vehicle-treated cells and cells treated with the microtubule-depolymerizing agent nocodazole or the actin-depolymerizing agent cytochalasin D. Nocodazole resulted in a virtually complete arrest of the movement of the punctate TRPM8-containing structures and a strong reduction in the total number of TRPM8 structures in the TIRF field (Fig. 1a–d). In contrast, speed was not affected in cells treated with cytochalasin D, although we found a slight reduction in total number of TRPM8-positive structures. These results indicate that the TRPM8-positive structures use microtubules as the principal track for rapid near-membrane intracellular movement.

### Dynamic co-localization of TRPM8 with LAMP1

**Figure 2:**
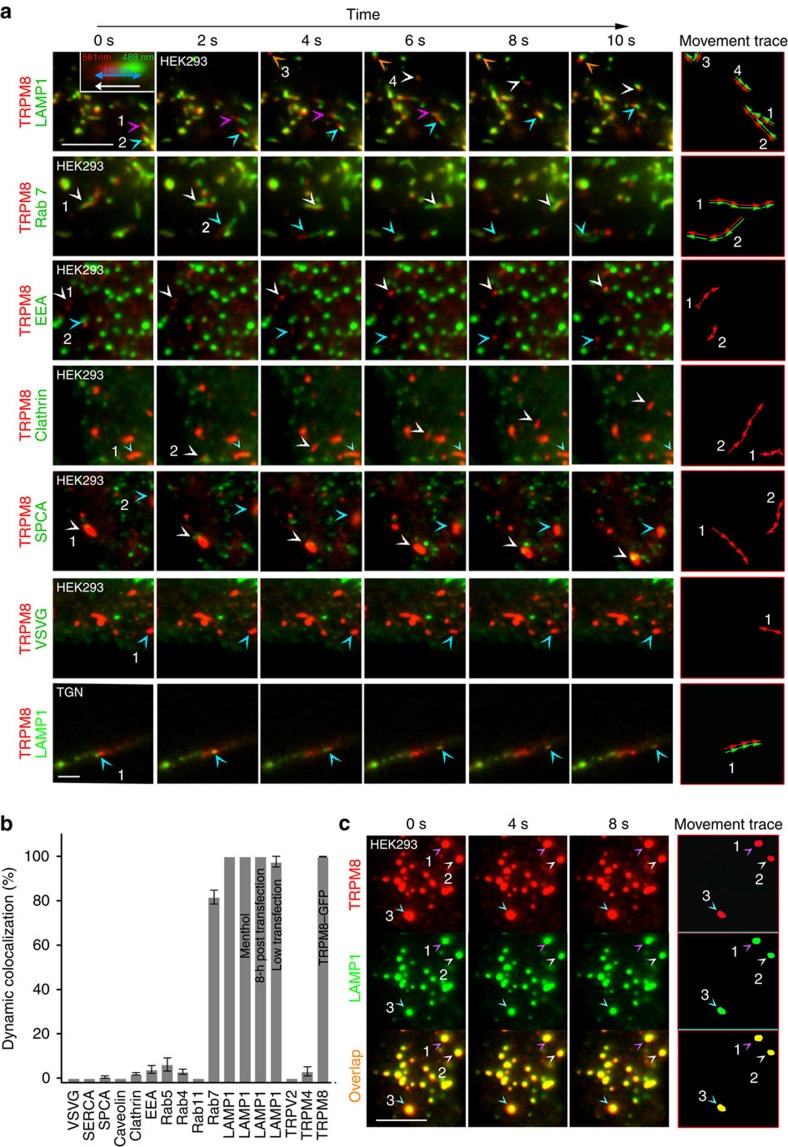
TRPM8 colocalizes with LAMP1 and Rab7 in mobile vesicles. (**a**) Dual-colour TIRF images at consecutive intervals showing the movement of TRPM8–mCherry (red) along with the indicated GFP-coupled marker proteins (in green) following co-expression in HEK293 cells or TGN. The arrowheads point at moving structures whose movement is tracked in the rightmost column. Scale bar, 5 μm. (**b**) Quantification of dynamic co-localization of TRPM8–mCherry with the indicated GFP-coupled proteins in HEK293 cells using the dynamic co-localization score (DCS). Data are shown as mean±s.e.m. (SPCA1, Caveolin, EEA1, Rab4, TRPV2, TRPM4 and TRPM8–GFP: *n*=6 cells; Clathrin and Rab4: *n*=8; Rab7, *n*=9; Rab11 and VSVG: *n*=12). DCS for LAMP1 was determined under control conditions (*n*=40), during a 5-min stimulation period with 50 μM menthol (*n*=6), at 8 instead of 16 h post transfection (*n*=8) and after reduction of the expression level of TRPM8–mCherry (*n*=6), as quantified in Supplementary Fig. 1. (**c**) Dual-colour TIRF images showing a high degree of static co-localization of TRPM8–mCherry and LAMP1–GFP following treatment with 30 μM of nocodazole. Scale bar, 5 μm.

To characterize the mobile TRPM8-positive structures, we co-expressed TRPM8–mCherry along with known markers of various cellular compartments tagged with green fluorescent protein (GFP), and quantified co-localization using the dynamic co-localization score (DCS; see methods^30^). As illustrated in Fig. 2a and Supplementary Movie 1, we observed strong dynamic co-localization of TRPM8–mCherry and LAMP1–GFP, yielding a DCS for LAMP1 of close to 100% (Fig. 2b). Dynamic co-localization of TRPM8 and LAMP1 was unaffected when TRPM8 was activated with menthol (Fig. 2b), and was also observed in neurites of TGN co-expressing TRPM8–mCherry and LAMP1–GFP (Fig. 2a). Following treatment with nocodazole, we found a high degree of overlap of static TRPM8–mCherry and LAMP1–GFP fluorescence, confirming the validity of our dynamic co-localization analysis (Fig. 2c). A high DCS (∼80%) was also observed for Rab7, whereas little or no dynamic co-localization (DCS<10%) was detected for all other tested markers (Fig. 2a,b).

### LAMP1-positive vesicles bring TRPM8 to the plasma membrane

Since LAMP1 and Rab7 are typically associated with endo-lysosomal structures^31^, we considered that the mobile TRPM8-positive punctate structures may be lysosomes involved in degradation of surplus TRPM8, and that their high abundance represents an artifact of the heterologous overexpression of the channel. Several results argue against this idea.

First, strong dynamic co-localization of TRPM8 and LAMP1 (DCS ∼100%) was observed (1) when the degree of TRPM8 overexpression was reduced by lowering the amount of complementary DNA (cDNA) used for transfection (0.33 μg instead of 1 μg; Supplementary Fig. 1; Fig. 2b); and (2) when cells were imaged after 8 instead of 16 h of transfection (Fig. 2b). These results indicate that co-localization of TRPM8 and LAMP1 is unrelated to the degree or timing of TRPM8 overexpression.

Second, we tested whether several other heterologously expressed TRP channels localize to the same mobile structures. We did not observe any significant dynamic co-localization of TRPM8 with either TRPM4 or TRPV2 (Fig. 2b; Supplementary Movie 2). Moreover, neither TRPM4 and TRPV2 nor the sensory TRP channels TRPA1 and TRPV1 showed any significant dynamic co-localization with LAMP1 (Supplementary Fig. 2). Notably, TRPM3, which also functions as a thermo- and chemosensitive ion channel in sensory neurons, did show a high level of dynamic co-localization with LAMP1 (Supplementary Fig. 2). These data indicate that localization in mobile, LAMP1-positive structures is a specific property of a limited number of TRP channels.

Third, the large majority of the TRPM8-positive structures (95.5±1.9%; *n*=10) or LAMP1-positive structure (93.3±2.5%; *n*=5) observed in the near-membrane zone were not stained by lysotracker red, a fluorescent dye that selectively stains acidic lysosomal compartments (Supplementary Fig. 3), indicating that the luminal pH of these structures is higher than that of classical lysosomes^32^. Note, however, that when observed in the epi-fluorescence mode, we observed sparse LT red-positive structures that contained TRPM8–GFP or LAMP1–GFP, which may represent TRPM8 and LAMP1 residing in acidic lysosomal structures in areas that are more distal from the plasma membrane. Comparable results were obtained using the dye pHrodo red dextran, which selectively accumulates in endocytotic compartments and whose intensity increases with acidification of the endocytotic structure (Supplementary Fig. 3). Moreover, when protein synthesis was blocked using cycloheximide, we did not observe any time-dependent decrease in the abundance or fluorescence intensity of the mobile TRPM8-positive structures, nor any decrease in functional TRPM8 (as observed through Fura-2-based calcium imaging) (Supplementary Fig. 4). Taken together, these results suggest that the pool of mobile TRPM8-positive vesicles represents a stable compartment rather than a lysosomal structure targeted for degradation.

**Figure 3:**
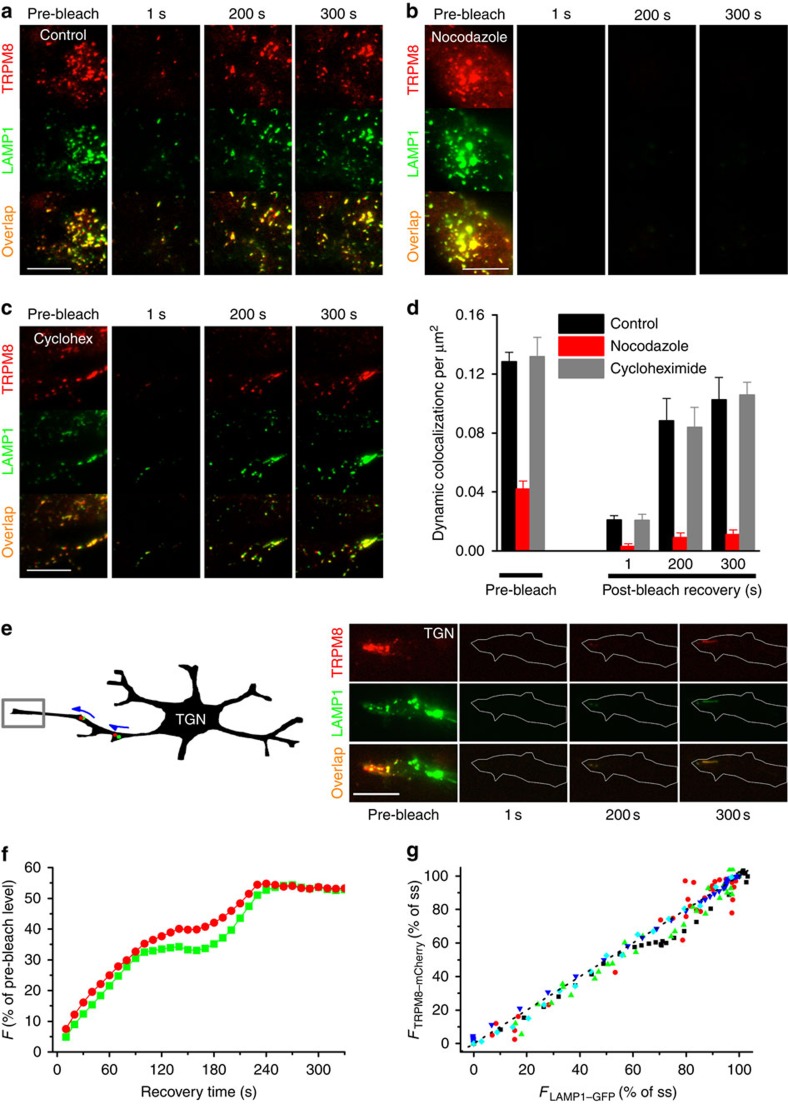
TRPM8 approaches the plasma membrane in LAMP1-containing vesicles. (**a**) Dual-colour TIRF images showing TRPM8–mCherry and LAMP1–GFP before bleaching of the near-membrane zone and at the indicated times of recovery. Scale bar, 10 μm. (**b**,**c**) Same experiment as **a** but in cells pretreated with 30 μM nocodazole (**b**) or 100 μM Cycloheximide (**c**). Scale bar, 10 μm. (**d**) Quantification of post-bleach recovery of the density of structures with dynamic co-localization of mCherry and GFP in HEK293 cells, normalized to cell surface area. *n*=6 cells for each condition. (**e**) Time-dependent recovery of TRPM8–mCherry and LAMP1–GFP following selective bleaching of the tip of the neurite of a TGN. Scale bar, 10 μm. (**f**) Representative example of the time course of recovery of TRPM8–mCherry and LAMP1–GFP fluorescence following photobleaching of a neurite tip. (**g**) Correlation of the recovery of TRPM8–mCherry and LAMP1–GFP fluorescence at neurite tips. Each data point represents the fluorescence in both channels at a single time point following bleaching, normalized to the steady-state fluorescence. Different symbols represent different neurons. The dotted line represents the line of equality.

To investigate whether the LAMP1-positive mobile structures transport TRPM8 towards the plasma membrane, we used TIRF Recovery after Photobleaching (TIR-FRAP), where we selectively bleached the fluorescently labelled TRPM8 and LAMP1 in close proximity of the glass–plasma membrane interface and followed their recovery after bleaching^33^ (Supplementary Fig. 5). Following bleaching, we observed substantial recovery of TRPM8–mCherry fluorescence in vesicular structures, and these vesicles were consistently LAMP1–GFP positive (Fig. 3a,d and Supplementary Movie 3). Similar results were obtained in the presence of cycloheximide, excluding a potential contribution of rapid local protein synthesis (Fig. 3c,d). In contrast, fluorescence recovery was almost completely inhibited by pretreatment with nocodazole (Fig. 3b,d). Comparable results were obtained in transfected TGN after 96 h in culture, at a time where these neurons develop long neurite extensions, and represent a simplified *in vitro* model for axonal transport^34^. When the neurite tip was bleached, substantial fluorescence recovery was observed via TRPM8- and LAMP1-positive vesicles transported from the cell body towards the tip of the neurite (Fig. 3e). While the time course of fluorescence recovery was variable between cells, recovery of LAMP1–GFP and TRPM8–mCherry was highly temporally correlated (Fig. 3f,g). Taken together, these results indicate that TRPM8- and LAMP1-positive mobile vesicles transport TRPM8 from within the cell towards the plasma membrane via microtubules.

### TRPM8 is associated with the vesicular SNARE protein VAMP7

**Figure 4:**
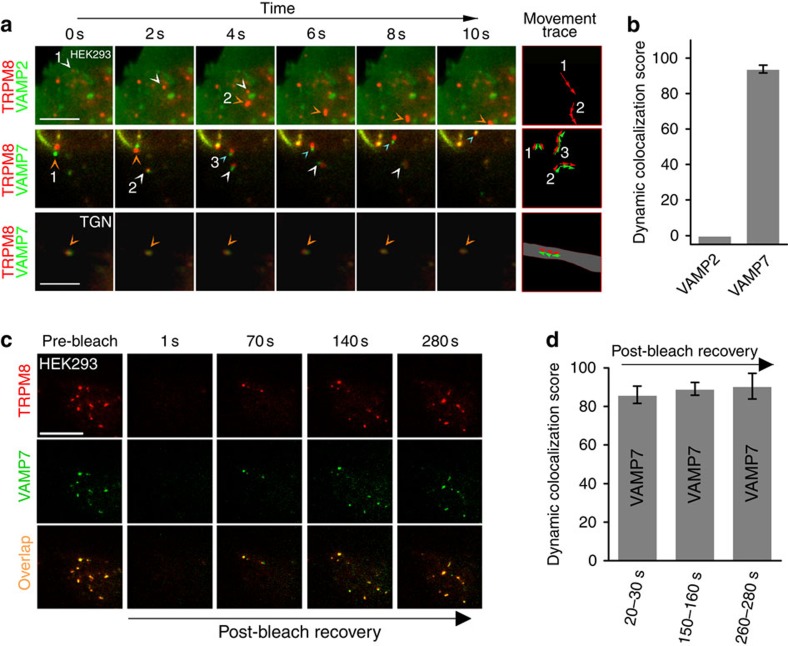
Dynamic co-localization of TRPM8 with the vesicular SNARE VAMP7. (**a**) Dual-colour TIRF images at consecutive intervals showing the movement of TRPM8–mCherry (red) along with VAMP2–GFP or VAMP7–GFP following co-expression in HEK293 cells or TGN. The arrowheads point at moving structures whose movement is tracked in the rightmost column. Scale bar, 5 μm. (**b**) Quantification of dynamic co-localization of TRPM8–mCherry with VAMP2–GFP or VAMP7–GFP in HEK293 cells. *n*=8 cells for each condition. (**c**) TIR-FRAP experiment illustrating the recovery of TRPM8 in VAMP7-positive vesicles. Scale bar, 10 μm. (**d**) Quantification of dynamic co-localization of TRPM8–mCherry and VAMP7–GFP at the indicated time points after bleaching in HEK293 cells. *n*=7 cells.

The vesicular SNARE protein VAMP7 is present in LAMP1-positive structures and mediates their fusion with the plasma membrane^35^. We found strong dynamic co-localization of TRPM8–mCherry with VAMP7–GFP, whereas VAMP2, which is typically associated with synaptic vesicles^36^, showed a clearly distinct distribution pattern (Fig. 4a,b).

**Figure 5:**
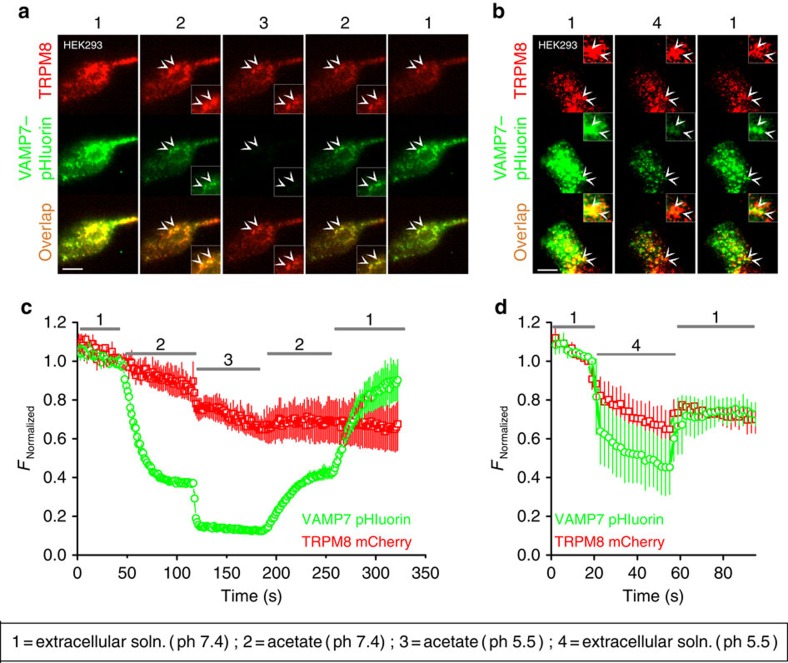
TRPM8 and VAMP7 colocalize in intracellular vesicles and at the plasma membrane. (**a**,**b**) Dual-colour TIRF images showing TRPM8–mCherry and VAMP7–pHluorin co-expressed in HEK293 cells, when perfused with the indicated extracellular solutions. The double arrowheads illustrate the co-expression of TRPM8–mCherry and VAMP7–pHluorin on the cell surface (inset). Scale bar, 10 μm. (**c**,**d**) Quantification of total TIRFM fluorescence intensity of TRPM8–mCherry and VAMP7–pHluorin during application of the indicated extracellular solutions. The fluorescent intensities are normalized to the intensity just before switching to solution 2 (**c**) or solution 4 (**d**). Data are shown as mean±s.e.m.

In TIR-FRAP experiments, we found dynamic co-localization of VAMP7–GFP and TRPM8 in mobile structures that repopulated the evanescent field following photobleaching (Fig. 4c,d; Supplementary Fig. 5; and Supplementary Movie 4). While these results demonstrate that TRPM8 and VAMP7 are together in structures that approach to the glass–plasma membrane interface, they do not indicate whether these structures are exclusively intracellular vesicles or also include vesicles that actually fuse with the plasma membrane, either fully or transiently^37^. To investigate this possibility, we expressed TRPM8–mCherry together with VAMP7 tagged with the ecliptic pHluorin at its luminal C terminus (VAMP7–pHluorin)^25^. Ecliptic pHluorin exhibits GFP-like green fluorescence at pH 7.4, but its fluorescence emission on excitation at 488 nm becomes almost completely quenched at pH ≤6.5. When cells were bathed in the standard NaCl-based extracellular solution (pH 7.4), TIRF imaging revealed a strong VAMP7–pHluorin signal in TRPM8-positive mobile structures, confirming our above-described findings that these structures are not acidic (Fig. 5a). To selectively quench the VAMP7–pHluorin in intracellular vesicles, cells were superfused with a solution containing Na-acetate (30 mM; pH=7.4), which causes acidification of intracellular compartments^38^. This resulted in a rapid quenching of VAMP7–pHluorin fluorescence to 50±2% of the pretreatment level, whereas TRPM8–mCherry remained stable (97±6% of pretreatment level) (Fig. 5a,c). Notably, the acetate treatment caused loss of pHluorin fluorescence in only a subset of VAMP7–pHluorin- and TRPM8–mCherry-positive structures, whereas other VAMP7–pHluorin- and TRPM8–mCherry-positive structures were insensitive to acetate treatment. Subsequent acidification of the extracellular solution to pH 5.5, in the continuous presence of acetate, resulted in almost total quenching of the VAMP7–pHluorin signal (18±3% of pretreatment level), whereas the TRPM8–mCherry signal sustained (83±6% of pretreatment level) (Fig. 5a,c). The quenching effects of acetate and low pH on the VAMP7–pHluorin signal were rapidly reversible on washout. Moreover, we found that rapid extracellular acidification of the NaCl-based solution from pH 7.4 to 5.5, resulted in an immediate and reversible loss of part of the VAMP7–pHluorin signal from loci where it colocalized with TRPM8–mCherry (Fig. 5b,d), whereas other VAMP7–pHluorin- and TRPM8–mCherry-positive structures were insensitive to extracellular acidification. These results indicate that TRPM8 colocalizes with VAMP7, both in non-acidic intracellular vesicles as well as in membrane regions that are accessible to the extracellular medium.

**Figure 6:**
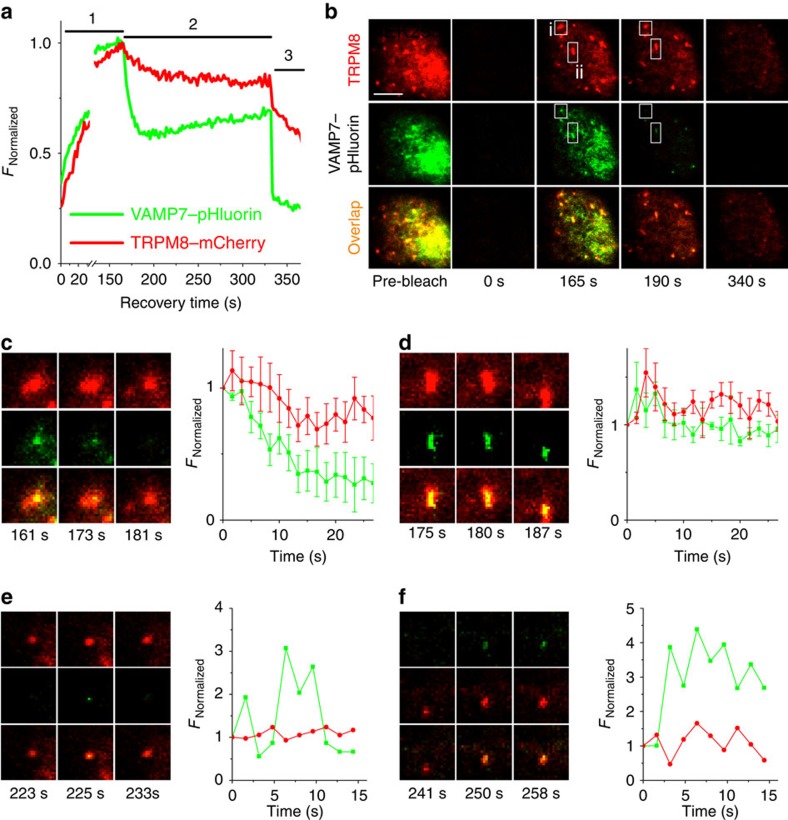
Vesicles containing TRPM8 and VAMP7 fuse with the plasma membrane. (**a**) Time course of total pHluorin and mCherry fluorescence following bleaching of the perimembrane area in normal extracellular solution at pH 7.4 (1), in acetate-containing solution at pH 7.4 (2), and in acetate-containing solution at pH 5.5 (3). (**b**) TIRF images obtained at the indicated time points. Scale bar, 5 μm. Boxed area indicate the regions analysed in **c** and **d**. (**c**) (left) TIRF images for boxed area (i) taken at the indicated time points. Frame dimension in **c**-**f** is 3 × 3 μm. (right) Mean fluorescence time course of structures that show similar acetate-induced quenching of green fluorescence, indicating localization of pHluorin in the lumen of an intracellular vesicle. (**d**) (left) TIRF images for boxed area (ii) taken at the indicated time points. (right) Mean fluorescence time course of structures that show similar insensitivity to acetate-induced quenching, indicating localization at the plasma membrane. (**e**,**f**) Individual vesicle fusion events observed in acetate-containing solution at pH 7.4, characterized by a sudden and transient (**e**) or more sustained (**f**) appearance of pHluorin fluorescence.

Next, we used TIR-FRAP to further distinguish distinct types of TRPM8-positive structures repopulating the evanescent field following photobleaching. We analysed fluorescence of VAMP7–pHluorin- and TRPM8–mCherry in concentric and equally spaced bands covering the entire footprint of the cells from the centre to the periphery^33^, and found that the time dependence of fluorescence recovery following photobleaching was relatively constant in the different bands (Supplementary Fig. 6). These results indicate that fluorescence recovery mainly occurs from inside the cell towards the plasma membrane rather than through lateral diffusion from non-bleached areas of the plasma membrane^33^. After a 150-s recovery period, the standard extracellular medium was replaced by acetate solution (pH 7.4) to specifically acidify intracellular compartments, allowing the discrimination of three distinct structures (Fig. 6a–f).

A first type included structures in which VAMP7–pHluorin was rapidly quenched by acetate, while TRPM8–mCherry fluorescence was sustained (Fig. 6c). We interpret these structures as intracellular vesicles containing pHluorin in their lumen.

A second type included structures where both TRPM8–mCherry and VAMP7–pHluorin remained stable in response to the acetate treatment (Fig. 6d). We interpret these as structures that are in contact with the extracellular space, and hence not acidified by acetate treatment.

Third, we also identified events indicative of exocytosis of individual TRPM8- and VAMP7-positive vesicles. In particular, under this experimental condition, we regularly (0.10±0.01 events per s per cell) observed incoming punctate structures that initially appeared only in the red (mCherry) channel, and suddenly also acquired green (pHluorin) fluorescence (Fig. 6e,f). The initial absence of green fluorescence indicates that these structures represent intracellular vesicles, whose lumen is acidified by the acetate, thus quenching the pHluorin. On fusion with the plasma membrane, the lumen of these vesicles becomes continuous with the extracellular solution at pH 7.4, resulting in a rapid de-quenching of VAMP7–pHluorin and appearance of green fluorescence. We observed events where the increase in green fluorescence was either transient (Fig. 6e) or more sustained (Fig. 6f). The former is in line with formation of a transient fusion pore between vesicle and plasma membrane, whereas the latter may represent sustained fusion^37^.

At the end of the experiment, residual VAMP7–pHluorin fluorescence was fully quenched by reducing the extracellular pH to 5.5 (Fig. 6a,b). Overall, these data provide direct evidence for the constitutive fusion of TRPM8- and VAMP7-positive vesicles with the plasma membrane.

Taken together, the above results establish that TRPM8 and VAMP7 are colocalized in intracellular vesicles that constitutively traffic to and fuse with the plasma membrane. In a recent study, Toro *et al*.^23^ reported that menthol stimulation of TRPM8-expressing F11 cells caused rapid recruitment of functional channels to the plasma membrane. In our experiments, we found that, like in HEK293 cells and TGN, TRPM8–mCherry expressed in F11 cells dynamically colocalizes with both LAMP1 and VAMP7 (Supplementary Fig. 7 and Supplementary Movie 5). However, we never observed any significant menthol-induced increase of TRPM8-fluorescence in near-membrane field in F11 cells (Supplementary Fig. 8). One possible explanation for this apparent discrepancy may lie in the analysis and interpretation of the TIRF measurements. In this respect, we note that Toro *et al*.^23^ performed TIRF imaging using a longer characteristic penetration depth (170 nm; compared with 90 nm in this study), and interpreted all increases in fluorescence in this evanescent field as fusion events. However, our results using pHluorin indicate that this interpretation is not fully correct, since a large fraction of the TRPM8-positive structures in the near-membrane remains intracellular (Figs 5 and 6).

### VAMP7 regulates the number of active TRPM8 channels

**Figure 7:**
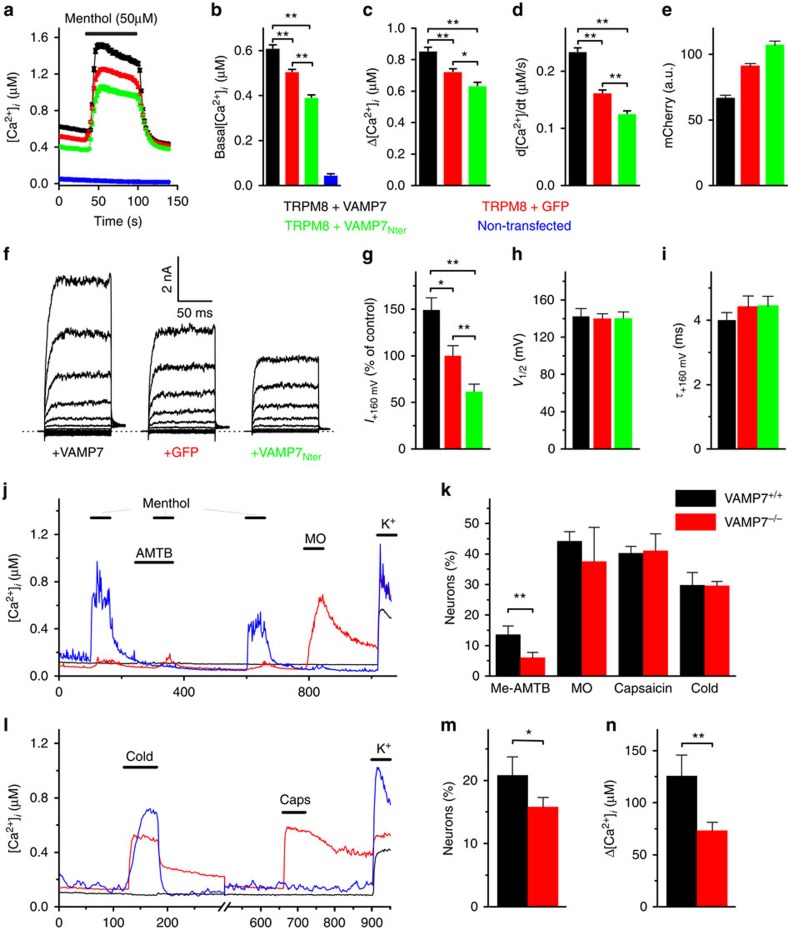
VAMP7 regulates plasma membrane expression of functional TRPM8. (**a**) Time course of [Ca^2+^]_*i*_ in HEK293 cells expressing TRPM8–mCherry along with either VAMP7–GFP (*n*=727), VAMP7_Nter_-IRES–GFP (*n*=383) or IRES–GFP (*n*=627) as well as non-transfected cells showing the effect of application of 50 μM menthol. (**b**) Comparison of basal [Ca^2+^]_*i*_. (**c**) Comparison of the menthol-induced rises in [Ca^2+^]_*i*_. (**d**) Comparison of the rates of the menthol-induced rise in [Ca^2+^]_*i*_. (**e**) Mean total mCherry fluorescence of TRPM8–mCherry-expressing cells when co-expressed either with VAMP7–GFP, IRES–GFP or VAMP7_Nter_-IRES–GFP. (**f**) Representative examples of whole-cell currents in HEK293 cells expressing TRPM8–mCherry along with VAMP7 (*n*=39), GFP (*n*=51) or VAMP7_Nter_ (*n*=27) in response to 100-ms voltage steps to potentials ranging from −120 to +160 mV (20 mV spaced) from a holding potential of 0 mV, followed by a voltage step to +60 mV. (**g**) Comparison of the steady-state TRPM8 current amplitudes at +160 mV, normalized to the mean amplitude in control (GFP-expressing) cells. (**h**) Voltage for half-maximal activation (*V*_1/2_), obtained from voltage steps as in panel **f**. (**i**) Exponential time constant for current relaxation at +160 mV. (**j**) Representative traces showing [Ca^2+^]_*i*_ in mouse VAMP7^−/−^ TGN in response to menthol (50 μM), mustard oil (MO; 50 μM) and high K^+^ (50 mM) solution. TRPM8-positive neurons (blue trace) could be identified by inhibition of the menthol response by AMTB (2 μM). (**k**) Percentage of total VAMP7^+/+^ and VAMP7^−/−^ TGN neurons that exhibited a response to menthol that was fully and reversibly blocked by AMTB (Me-AMTB) or responses to MO, capsaicin (1 μM) and cold (10 °C). In total, 1,372 VAMP7^+/+^ and 1,057 VAMP7^−/−^ neurons were investigated from 6 animals per genotype. (**l**) Representative traces showing [Ca^2+^]_*i*_ in mouse VAMP7^−/−^ TGN in response to cold, capsaicin and high K^+^ solution. (**m**) Percentage of the capsaicin-insensitive VAMP7^+/+^ and VAMP7^−/−^ TGN neurons that exhibited a response to cold. (**n**) Average cold response in capsaicin-insensitive VAMP7^+/+^ and VAMP7^−/−^ TGN neurons. Significance was tested using one-way ANOVA with Tukey's *post hoc* test (**b**–**e** and **g**-**i)**, two-sample independent *t*-test (**n**) or Fisher's exact test (**m**). *P<0.05, **P<0.01.

The above findings raised the possibility that VAMP7 regulates the abundance of TRPM8 at the plasma membrane, as a vesicular SNARE involved in the exocytosis of TRPM8-positive vesicles. To investigate this, we first performed Fura-2-based calcium imaging in HEK293 cells expressing TRPM8–mCherry along with either wild-type (WT) VAMP7, cytosolic GFP (control) or the N terminus of VAMP7 (VAMP7_Nter_). The VAMP7_Nter_, also known as Longin domain, acts as a specific inhibitor of VAMP7 function by binding to the SNARE domain and thus preventing association with t-SNAREs^39^,^40^. At room temperature, TRPM8-expressing cells typically show a high basal [Ca^2+^]_*i*_ compared with non-transfected HEK293 cells, due to significant TRPM8 activity at temperatures below 30°. In cells co-expressing WT VAMP7, we measured a significantly higher basal [Ca^2+^]_*i*_ compared with cells co-expressing cytosolic GFP, whereas cells co-expressing VAMP7_Nter_ exhibited a reduced basal [Ca^2+^]_*i*_ (Fig. 7a,b). Moreover, responses to menthol were increased in cells expressing WT VAMP7 and decreased in cells expressing VAMP7_Nter_ (Fig. 7a,c,d). To examine whether the different calcium responses could be due to differences in protein expression, we quantified TRPM8–mCherry fluorescence as a direct measure of total cellular TRPM8. Surprisingly, average cellular TRPM8–mCherry fluorescence in cells expressing WT VAMP7 was ∼25% lower than in control cells and ∼40% lower than in cells expressing VAMP7_Nter_ (Fig. 7e). Based on this result, we can exclude that the observed differences in TRPM8-mediated intracellular Ca^2+^ signals merely reflect changes in protein expression of TRPM8. Instead, the combination of lower total TRPM8–mCherry signal with higher functional TRPM8-mediated responses in the VAMP7-overexpressing cells suggests that VAMP7 enhances functional incorporation in the plasma membrane.

Next, we performed patch-clamp experiments to directly assess the influence of VAMP7 on functional TRPM8 expression and gating. Whole-cell currents were measured at room temperature, yielding typical voltage-dependent, outwardly rectifying currents. Compared with the GFP control, the current amplitude was significantly increased by co-expression of WT VAMP7, and decreased by VAMP7_Nter_ (Fig. 7f,g). Importantly, WT VAMP7 and VAMP7_Nter_ did not affect the voltage for half-maximal activation (*V*_1/2_) or the exponential time constant for current relaxation at +160 mV, suggesting that channel gating was not altered (Fig. 7h,i).

We also evaluated whether other TRP channels exhibit VAMP7-dependent regulation, and focused on three other sensory channels, TRPV1, TRPA1 and TRPM3. Using TIRF microscopy, we found a high degree (86±6%) of dynamic co-localization of TRPM3–mCherry with VAMP7–GFP, whereas less than 20% of TRPA1-positive structures showed dynamic co-localization with VAMP7, and no co-localization was observed for TRPV1 (Supplementary Fig. 9). The differential co-localization of these TRP channels was mirrored by differential modulation of their functional responses by VAMP7. Indeed, we measured a robust potentiation of the TRPM3-mediated response to the agonist pregnenolone sulphate by WT VAMP7, as well as a strong inhibition by VAMP7_Nter_ (Supplementary Fig. 9). In contrast, in the case of TRPA1 and TRPV1 we did not observe any significant effect of WT VAMP7 on calcium responses to their respective agonists mustard oil and capsaicin; VAMP7_Nter_ had a mild inhibitory effect on TRPA1 (Supplementary Fig. 9).

It is well-established that TRPM8 is highly expressed in a subset of sensory neurons^5^,^6^,^41^. Using quantitative real-time PCR, we additionally detected prominent expression of VAMP7, LAMP1 and Rab7 in isolated dorsal root ganglia (DRG) and TG (Supplementary Fig. 10). To evaluate whether VAMP7 affects functional expression of endogenous TRPM8 in sensory neurons, we performed intracellular Ca^2+^ measurements in TGN isolated from VAMP7^+/+^ (WT) and VAMP7^−/−^ mice. First, to specifically identify TRPM8-expressing neurons, we applied three successive menthol stimuli (50 μM) and included the TRPM8-specific blocker (*N*-(3-Aminopropyl)-2-[(3-methylphenyl)methoxy]-*N*-(2-thienylmethyl)benzamide hydrochloride; 2 μM) during the second menthol stimulus. We found that ∼14% of WT TGNs exhibited a response to menthol that was fully and reversibly blocked by AMTB. Neurons exhibiting this response profile were considered TRPM8^+^ neurons. Importantly, the fraction of TRPM8^+^ neurons was significantly reduced to 6% in VAMP7^−/−^ mice (Fig. 7j,k). Note that, both in WT and VAMP7^−/−^ mice, we found neurons that exhibited AMTB-insensitive responses to menthol (Fig. 7j). These responses occurred exclusively in the subset of neurons that also exhibited robust responses to the TRPA1-agonist mustard oil, and thus most likely reflect the known moderate agonistic action of menthol on TRPA1 (ref. 42). We found that ∼40% of the WT neurons responded to mustard oil, and the percentage mustard oil responders was similar in VAMP7^−/−^ mice. Likewise, we did not find differences between WT and VAMP7^−/−^ neurons with respect to the fraction of responders to the TRPV1 agonist capsaicin. These data indicate that function of both TRPV1 and TRPA1 is not significantly affected by the VAMP7 deficiency.

Next, we compared TGN from WT and VAMP7^−/−^ mice with respect to their responsiveness to a cold stimulus. In line with earlier work^43^,^44^, we found that approximately one fourth of WT neurons responded to cooling of the bath solution to 10 °C, and the overall percentage of cold-responsive neurons was not significantly different in VAMP7^−/−^ mice (Fig. 7k,l). At first sight, the unaltered fraction of cold-sensitive neurons may seem contradictory to the substantial reduction in TRPM8-mediated responses in the VAMP7^−/−^ mice as identified based on menthol and AMTB sensitivity (Fig. 7k). However, it should be noted that TRPM8 underlies only a small fraction of cold responses in these neurons, and that various other mechanisms can mediate cold sensitivity, which include TRPA1-dependent and TRPM8/TRPA1-independent pathways^1^. Earlier work has provided evidence that TRPM8-mediated cold-responses in mouse sensory neurons are mainly confined to capsaicin-insensitive sensory neurons, whereas TRPA1-dependent responses mainly occur in capsaicin-sensitive neurons^44^. We therefore specifically examined cold sensitivity in those cells that did not respond to capsaicin (Fig. 7l). Importantly, in this subset of neurons, we observed a significant reduction in the both the number of cells that showed a detectable cold response as well as in the amplitude of the cold-induced calcium increase (Fig. 7l–n). Taken together, these results indicate that VAMP7 deficiency leads to reduced TRPM8 activity in sensory neurons.

### Cold avoidance and cold hypersensitivity in VAMP7^−/−^ mice

Finally, we investigated whether VAMP7 deficiency leads to impaired TRPM8-dependent sensory responses *in vivo*. Earlier work has established that genetic ablation or pharmacological inhibition of TRPM8 leads to prominent deficiencies in various aspects of cold sensing^2^,^3^,^4^,^7^, including impaired avoidance of temperatures in the innocuously cold temperature range between 15 and 25 °C and reduced behavioural responses to TRPM8-activating cooling agents such as icilin. To evaluate TRPM8 function *in vivo*, we compared WT and VAMP7^−/−^ mice in the thermal gradient and the icilin-induced cold hypersensitivity tests.

**Figure 8:**
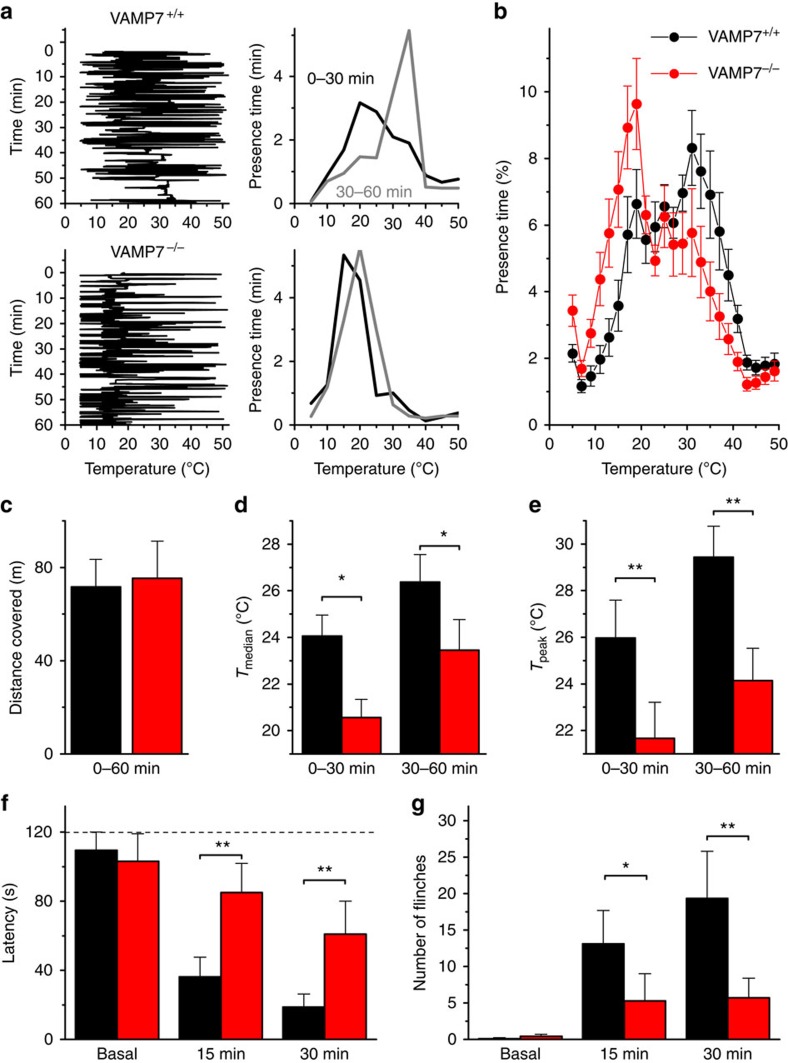
Reduced TRPM8 functionality in VAMP7^−/−^ mice *in vivo.* (**a**) Representative experiments depicting the positions of a VAMP7^+/+^ and a VAMP7^−/−^ mouse on a thermal gradient plate during a 1-h experiment (left) and the corresponding presence time in the different temperature zones during the first and second 30-min periods (right). (**b**) Mean occupancy of the thermal gradient during 1 h by VAMP7^+/+^ (n =15) and VAMP7^−/−^ (*n*=14) mice. (**c**) Mean distance covered on the thermal gradient by VAMP7^+/+^ and VAMP7^−/−^ mice. (**d**) Comparison of the median temperature (*T*_median_) in both genotypes during the first and second 30-min periods. (**e**) Comparison of the peak temperature (*T*_peak_) in both genotypes during the first and second 30-min periods. (**f**) Latency of the first nocifensive response on a 1 °C cold plate before (basal) and at 15 and 30 min after injection of left hind paw with 10 μl of a 5 mM icilin solution. Cut-off time was set at 120 s (dashed line). (**g**) Number of flinches on the 1 °C cold plate before (basal) and at 15 and 30 min after injection of the left hind paw. Significance was tested using two-way repeated measures ANOVA with Tukey's *post hoc* test. *P<0.05, **P<0.01.

In the thermal gradient assay, mice were allowed to move freely on a flat temperature gradient plate ranging from 5 °C to 50 °C, and their position on the gradient was tracked during 60 min (Fig. 8a,b). Mice of both genotypes covered the same distance on the gradient (Fig. 8c), in line with earlier behavioural tests showing that VAMP7^−/−^ mice exhibit normal overall locomotor activity in the open-field test^28^. There was also no significant difference in avoidance of the noxiously hot end of the gradient (>43 °C), where WT mice and VAMP7^−/−^ mice spent 5.3±0.7% and 4.3±0.7% of the time, respectively (*P*=0.33). This is also in general agreement with published data showing unaltered heat avoidance behaviour of VAMP7-deficient mice in the hot-plate assay^28^.

In line with the literature, we found that WT mice extensively explore the gradient, especially in the non-noxious temperature range (15–40 °C), and gradually reach a peak ‘preferred' temperature (*T*_peak_) of ∼30 °C (Fig. 8a,b,d). In comparison, VAMP7^−/−^ mice showed a systematic and highly significant preference for colder temperatures, as evidenced by values for both *T*_peak_ and the median occupied temperature (*T*_median_) that were on average 3–4 °C lower than for WT (Fig. 8d,e). Overall, the VAMP7^−/−^ mice spent 61.2±5.3% of the first hour in the temperature range ≤25 °C, compared with only 43.4±5.4% in the WT animals (*P*=0.027). Taken together, these data indicate that absence of VAMP7 leads to a specific deficit in the avoidance of innocuously cold temperatures *in vivo*.

Intraplantar injection of icilin causes a strong decrease in withdrawal latency from a cold plate, and this icilin-induced cold hypersensitivity is completely abolished in TRPM8-deficient mice^4^,^45^. We tested the paw withdrawal latency and pain behaviour on a 1 °C cold plate, before and at two time points following injection of icilin in one hind paw of WT and VAMP7^−/−^ mice. We observed a sharp decrease in withdrawal latency in WT mice at both 15 and 30 min following icilin injection. Importantly, the icilin-induced reduction in withdrawal latency was remarkably and significantly blunted in VAMP7^−/−^ mice (Fig. 8f). Likewise, the number of flinches on the cold plate was significantly lower in VAMP7^−/−^ mice compared with WT (Fig. 8g). Taken together, these data indicate that absence of VAMP7 results in a strong deficit in icilin-induced cold hypersensitivity.

## Discussion

TRPM8 plays a key role in thermosensation by the somatosensory system, where its activation by cold or cooling chemical agents evokes a depolarizing current in a subset of cold-activated sensory neurons^1^. The size of the depolarizing current, and accordingly the intensity of the cold sensation, critically depends on the number of functional TRPM8 channels at the plasma membrane. In the present work, we provide important novel insights into the molecular and cellular mechanisms that underlie the transport of TRPM8 to the plasma membrane. We provide evidence that TRPM8 is transported to the plasma membrane via microtubules and using unconventional transport vesicles that contain LAMP1 but are not acidic. Moreover, we provide evidence that the vesicular SNARE VAMP7 participates in the fusion of these vesicles with the plasma membrane. The importance of VAMP7 is further corroborated (1) by the finding that a dominant negative VAMP7 construct reduces the number of active TRPM8 channels in the plasma membrane, (2) by the reduced TRPM8 activity in sensory neurons from VAMP7^−/−^ mice and (3) by the specific deficiencies in TRPM8-dependent cold avoidance and icilin-induced cold hypersensitivity in these mice.

Our observation that TRPM8 is abundantly present in VAMP7- and LAMP1-containing vesicles that approach and fuse with the plasma membrane is surprising but not unprecedented. Although LAMP1 is generally considered as a lysosomal marker^31^, it does not play a major role in maintenance of major lysosomal traits (low pH, activity of lysosomal enzymes, lysosomal density or shape)^46^. Instead, LAMP1 seems to be involved in heterotypic fusion of intracellular structures^47^ and movement of endosomal structures along microtubules^48^,^49^. In fact, trafficking of LAMP1 to the plasma membrane has been recognized long ago^50^, and LAMP1-positive compartments are believed to represent not only compartments for degradation but also secretory lysosomes^51^. Recently, evidence was presented that TRPML1, a TRP channel that is generally associated with lysosomes, can actually reach the plasma membrane of muscle cells on fusion of vesicles containing TRPML1, VAMP7 and LAMP1 (ref. 52). Fusion of these vesicles and thus appearance of functional TRPML1 at the plasma membrane was hardly observed under basal conditions, but became prominent following maneuvers that damage the cell membrane, and represents a mechanism for membrane repair^52^. In contrast, our present results suggest that fusion of VAMP7- and TRPM8-containing vesicles represents a mechanism for the constitutive transport of TRPM8 to the plasma membrane.

We also tested several other TRP channels for dynamic co-localization with LAMP1 and VAMP7 in HEK293 cells. In these experiments, we did not find evidence for any significant co-localization of the other sensory TRP channels TRPV1 and TRPA1 with LAMP1 or VAMP7. However, in the case of TRPM3 we found strong dynamic co-localization with both LAMP1 and VAMP7. Moreover, TRPM3 activity in HEK293 cells was strongly enhanced by WT VAMP7 and inhibited by dominant negative VAMP7. These findings suggest that VAMP7-dependent transport is not unique for TRPM8 but may be utilized by a subset of TRP channels, including TRPM8, TRPM3 and TRPML1.

Contrary to a recent study^23^, we were not able to find any evidence for increased trafficking of TRPM8 towards the plasma membrane in response to the TRPM8 agonist menthol, despite the fact that this stimulus consistently provoked a robust intracellular Ca^2+^ rise. At present, we do not have a full explanation for the apparent discrepancy between our results and those of Toro *et al*.^23^ However, we would like to point out that Toro *et al*.^23^ interpreted increases in TRPM8–GFP fluorescence in the TIRF field as fusion events, whereas their approach does not allow discriminating between TRPM8–GFP on the plasma membrane and subplasmalemmal TRPM8. Indeed, our present results indicate that the majority of TRPM8 molecules observed under TIRF actually colocalizes with VAMP7–pHluorin in intracellular vesicles. Moreover, we note that the reported threefold increase in plasma membrane TRPM8 on menthol stimulation^23^ appears incompatible with several published observations, including (1) the very rapid (subsecond) onset and washout of the agonist effect of menthol in intact cells, excised patches and lipid bilayers; (2) the general observation that effects of menthol on TRPM8-mediated currents can be fully described by an effect on channel gating; and (3) the fact that prolonged menthol stimulation leads to a reduction rather than a sensitization of subsequent menthol TRPM8-mediated responses^12^,^13^,^14^,^15^,^16^,^17^,^53^. Nonetheless, our results do not exclude that transport of TRPM8 may be up- or downregulated under certain (patho)physiological conditions in a VAMP7-dependent or -independent manner.

Isolated TGN from VAMP7^−/−^ mice exhibited reduced TRPM8-dependent responses, whereas responses to the TRPA1-agonist mustard oil or the TRPV1 agonist capsaicin were not altered. The residual TRPM8 activity in sensory neurons from VAMP7^−/−^ mice indicates that TRPM8 may reach the plasma membrane, via other transport mechanisms or that related VAMP proteins may partially compensate for loss of VAMP7 (ref. 28). *In vivo*, we found that VAMP7^−/−^ mice show impaired avoidance to cooler temperatures and reduced icilin-induced cold hypersensitivity. These phenotypes are similar but less pronounced than what has been described in the TRPM8^−/−^ animals^4^. These findings provide strong evidence that VAMP7 is required for the normal function of TRPM8 as a cold sensor involved in cold sensing and cold hypersensitivity, but also confirm the existence of VAMP7-independent transport mechanisms.

VAMP7 is widely distributed in the adult mammalian nervous system, in particular in vesicles and tubules in axonal and dendritic outgrowths, where it concentrates into the leading edge of the growth cone^54^. VAMP7-containing vesicles have been shown to move on microtubules from the cell soma to the periphery^25^. At hippocampal synapses, VAMP7 was found to be mainly present on a resting pool of synaptic vesicles and to modulate non-evoked, spontaneous exocytosis of these vesicles^55^. Our present findings indicate that (1) VAMP7 marks specific vesicles that transport TRPM8 to the plasma membrane, including neurites of sensory neurons; (2) this transport requires intact microtubules; and (3) VAMP7 is involved in constitutive fusion of these vesicles with the plasma membrane. Earlier work has shown that VAMP7^−/−^ mice are viable and show no striking developmental or neurological defects (including unaltered responses to painfully hot and electrical stimuli), but exhibit slightly increased anxiety^28^. Interestingly, the same study also revealed that VAMP7^−/−^ mice have a slightly lower core body temperature than WT mice^28^. Our current findings suggest that reduced TRPM8 activity could contribute to hypothermia in VAMP7^−/−^ mice. Indeed, inhibition of TRPM8 activity reduces cold defense and cold avoidance mechanisms, which may ultimately result in mild hypothermia^7^,^8^. Targeting VAMP7 function may represent a new opportunity to reduce TRPM8 activity in patients with cold allodynia or other TRPM8-related pain syndromes.

## Methods

### Animals and behavioural experiments

All experiments were performed using 12–16-week-old female WT and VAMP7^−/−^ mice^28^ on a C57BL/6 N background. Animals were housed in a conventional facility at 21 °C on a 12-h light–dark cycle with chow diet and water available *ad libitum*. All experiments involving animals were approved by the KU Leuven Ethical Committee Laboratory Animals under project number P192/2014. All behavioural experiments were performed by an experimenter blind to genotype.

In the thermal gradient test, mice were allowed to freely move on a 120-cm-long/18-cm-wide metallic plate having temperatures of 5 and 50 °C at the extreme ends (Bioseb—*In Vivo* Research Instruments)^56^. Mice were placed on the plate for 60 min and their free movement was monitored using a digital camera and tracking software. Animals were habituated to the plate for 60 min for 2 days preceding the experiment.

In the icilin-induced cold hypersensitivity test, mice were placed on a 1 °C cold plate, and the latency to the first brisk hind paw lift or flicking/licking of the hind paw was measured, with a cut-off time of 120 s. Latency was measured before and at 15 and 30 min after injection of left hind paw with 10 μl of a solution containing 80% DMSO/20% PBS supplemented with 5 mM icilin^4^.

### Cell lines

HEK293 cells (from American Type Culture Collection) were grown in Dulbecco's modified Eagle's medium (DMEM) that contained 10% (v/v) human serum, 2 mM L-glutamine, 2 U ml^−1^ penicillin and 2 mg ml^−1^ streptomycin at 37 °C in a humidity-controlled incubator with 10% CO_2_. F11 cells (from American Type Culture Collection) were grown in Ham's F12 medium (Invitrogen) that contained 10% (v/v) foetal bovine serum (Invitrogen), 1.5 mM *L*-hydroxyproline (Sigma) and 1% Glutamax (Invitrogen), at 37 °C in a humidity-controlled incubator with 10% CO_2_. HEK293 and F11 cells were transfected with cDNA (1 μg, unless mentioned otherwise) encoding the protein or proteins of interest using the Mirus 293 Transfection Reagent (Mirus Corporation, Madison, USA). For TIRF experiments, HEK293 or F11 cells were reseeded on 25 mm poly-*L*-lysine (PLL)-coated glass coverslips after 4 h of transfection, and kept under identical culture conditions for 12–18 h before imaging. For calcium imaging and patch-clamp experiments, cells were transfected overnight and then reseeded on PLL-coated coverslips for 2–6 h before recording.

### Mouse sensory neurons

For TIRF measurements, 12–16-week-old WT female mice were killed in a CO_2_ chamber and the trigeminal ganglia were excised under a microscope. Isolated ganglia were washed in PBS (Invitrogen) and collected in cold Leibowitz medium (L15; Invitrogen). Ganglia were cut into small pieces and incubated (37 °C; 5% CO_2_) for 45 min in warm DMEM containing 0.025% collagenase (type IA; Sigma). Tissue was gently triturated with a fire-polished glass pipette, and the suspension was centrifuged at 200*g* for 8 min. The obtained pellet was resuspended in culture medium with the following composition: DMEM/F12 medium (1:1) with 1% Glutamax supplemented with 10% foetal calf serum, 100 ng ml^−1^ NGF-7 S mouse and 100 μg ml^−1^ penicillin/streptomycin. Cells were plated on 25 mm glass coverslips covered with PLL and kept in a humidified atmosphere (37 °C; 5% CO_2_). One hour after plating, 2 ml of culture medium was added to each dish. Neurons were grown for 48 h before transfection with cDNA encoding protein or proteins of interest using the TransIT-Neural Transfection Reagent (Mirus Corporation). TGNs were kept under identical culture conditions for 48 h after transfection, before TIRF imaging.

For calcium imaging, 12–16-week-old WT and VAMP7^−/−^ female mice were killed in a CO_2_ chamber and the trigeminal ganglia were excised under a microscope. Isolated ganglia were washed in 10% foetal calf serum Neurobasal A Medium (basal medium, Invitrogen) and further digested for 40 min at 37 °C with a mixture of collagenase at 1 mg ml^−1^ (Gibco) and dispase at 2.5 mg ml^−1^ (Gibco). The digested tissue was washed twice with basal medium and mechanically dissociated by passage through syringes fitted with sequentially increasing needle gauges. Neurons were then plated on poly-*L*-ornithine/laminin-coated glass bottom chambers (Fluorodish WPI) and cultured overnight (12–16 h) in a humidified atmosphere (37 °C; 5% CO_2_) in B27-supplemented neurobasal A medium containing 2 ng ml^−1^ glial-derived neurotrophic factor (Invitrogen) and 10 ng ml^−1^ neurotrophin-4 (Peprotech).

### TIRF microscopy

Cells on 25-mm glass coverslips were placed in a custom-made chamber and imaging experiments were performed at 25 °C. Experiments with HEK293 and F11 cells were performed with a HEPES-buffered saline that contained (in mM): 150 NaCl, 5 MgCl_2_, 3CaCl_2_ and 10 HEPES. The pH of this solution was set at 7.4 using NaOH, unless mentioned otherwise. To acidify intracellular compartments in experiments with VAMP7–pHluorin, we used an extracellular solution that contained (in mM) 90 NaCl, 2 CaCl_2_, 1 MgCl_2_, 60 Na-acetate and 10 HEPES (pH 7.4 or 5.5 with NaOH). Experiments with TGN were performed with a HEPES-buffered saline that contained (in mM): 140 NaCl, 5 KCl, 1 MgCl_2_, 1.5 CaCl_2_, 10 Glucose monohydrate and 10 HEPES (pH 7.4 with NaOH).

TIRF images were acquired using a through-the-lens TIRF system that was built around an inverted Axio Observer.Z1 microscope equipped with a × 100 oil objective numerical aperture (NA)=1.45 (Zeiss), a Hamamatsu Orca-R^2^ camera, and 488-nm and 561-nm lasers. Time series of images at 500-ms intervals were recorded. Constant focus was maintained using the Definite Focus module (Zeiss). In the experiments involving dual channel recording, for each image in a time series both 488-nm and 561-nm lasers were used consecutively (having a time delay of 150 ms between them). The TIRF angle was set for both the lasers to achieve an evanescent field with a characteristic penetration depth (that is, the distance in the *z* direction over which the intensity declines e-fold) of 90 nm. Since the 488-nm and 561-nm laser excitation and fluorescence-recording epochs were sequential, rapidly moving structures containing both mCherry-fused and GFP-fused marker proteins were observed as dual coloured spots, where the red fluorescence preceded the green fluorescence. We used the following marker proteins: clathrin^57^ and caveolin^58^ as markers for endocytic structures; EEA1 (ref. 59), Rab4 and Rab5 (refs 60, 61) for early endosomes; Rab11 for recycling endosomes^62^; Rab7 for late endosomes^63^; LAMP1 for lysosomes^31^; the secretory pathway Ca^2+^-ATPase (SPCA1) for Golgi^64^; the sarco/endoplasmic reticulum Ca^2+^-ATPase (SERCA2) for endoplasmic reticulum^65^; and vesicular stomatitis virus G-protein, a widely studied marker for the secretory pathway^66^. SPCA1 and SERCA2 contained GFP coupled to their N termini, while all other marker proteins had GFP coupled to their C termini.

Analysis fluorescence intensity and of the speed of moving structures was performed with AxioVision 4.8 digital image processing software (Zeiss). Quantification of particle size and number was performed with Imaris software (Bitplane). To determine whether two fluorescently labelled molecules are present in the same mobile vesicular structure, we performed dynamic co-localization analysis^30^. This method is based on the postulate that synchronized movement over several frames of a movie is a much stronger evidence for object co-localization than pixel co-localization in still images analysis^30^. For dynamic co-localization analysis, we only considered mobile TRPM8–mCherry-positive structures whose trajectory could be tracked during at least five consecutive frames, and then evaluated whether correlated mobility of GFP fluorescence occurred. The analysis resulted in a DCS, which was defined as the ratio of the number of mobile TRPM8–mCherry-positive structures with correlated GFP fluorescence over the total number of mobile TRPM8–mCherry-positive structures, expressed in %. As a positive control, we co-expressed TRPM8–mCherry and TRPM8–GFP (Supplementary Fig. 1; Supplementary Movie 6), and obtained a DCS of 100%.

All supplementary movies provided with this manuscript are shown at a frame rate of 5 frames per s.

### RT–PCR

Mice were killed in a CO_2_ chamber and the trigeminal and DRG were carefully dissected^41^. DRG from the different segments of the spinal cord were pooled. Total RNA from trigeminal ganglia and pooled DRG was extracted with RNeasy Mini Kit (Qiagen), following the manufacturer's protocol. Quality and concentration of the extracted RNA was assessed using the Experion RNA StdSens Analysis Kit (Bio-Rad), and samples with RQI values <6 were discarded. Further, cDNA was synthesized with the extracted RNA using Ready-To-Go You-Prime First-Strand Beads (GE Healthcare). Quantitative reverse transcription–PCR (RT–PCR) was then performed following the protocol described before using 2 μl cDNA template, Universal TaqMan MasterMix (2 × concentrated, Life Technologies), TaqMan assay (20 × concentrated, Life Technologies). The used primers are listed in Supplementary Table 1. Non-template controls were used as negative controls in every experiment. PGK1 and HPRT1 were used as endogenous controls. Data are expressed as relative expression of detected mRNA normalized to PGK1.

### Measurement of intracellular Ca^2+^ concentration ([Ca^2+^]_*i*_)

Changes in [Ca^2+^]_*i*_ in HEK293 cells and TGN were monitored using ratiometric Fura-2-based fluorimetry. Cells were loaded with 2 μM Fura-2AM-ester (Alexis Biochemicals) for 30 min. Fluorescence was measured during repetitive illumination at 340 and 380 nm using the filter-based MT-10 illumination system and xcellence pro software (Olympus). Absolute calcium concentrations were calculated from the ratio of the fluorescence signal at both wavelengths was calculated^67^.

### Whole-cell patch-clamp recordings

After 16 h of transfection, currents in HEK293 cells were recorded in the whole-cell configuration of the patch-clamp technique using an EPC-9 amplifier and Patchmaster software (HEKA Elektronik). Data were sampled at 5 kHz and digitally filtered off-line at 2 kHz. Between 60 and 90% of the series resistance was compensated, reducing voltage errors to <10 mV. The standard intracellular solution for whole-cell measurements contained: 150 mM NaCl, 3 mM MgCl_2_, 5 mM EGTA and 10 mM HEPES at pH 7.2. The standard extracellular solution contained: 150 mM NaCl, 6 mM CsCl, 1 mM MgCl_2_, 1.5 mM CaCl_2_, 10 mM glucose and 10 mM HEPES at pH 7.4.

To determine the potential for half-maximal activation (*V*_1/2_), steady-state current–voltage relations measured at the end of 100-ms voltage steps were fitted with the product of a Boltzmann equation and a linear function, as described earlier^12^. To determine the time constant of current relaxation, a monoexponential function was fitted to the current during the voltage step to +160 mV.

### Chemicals

Menthol, mustard oil, AMTB (*N*-(3-Aminopropyl)-2-[(3-methylphenyl) methoxy ]-*N*-(2-thienylmethyl)benzamide hydrochloride), Nocodazole, cytochalasin D and cycloheximide were obtained from Sigma-Aldrich. pHrodo Red dextran and lysotracker Red were purchased from Invitrogen.

### Statistics

Data analysis was performed using Origin 8.6 (OriginLab Corporation). Group data are expressed as mean±s.e.m. The Shapiro–Wilk test was used to test the normality of the data. Student's unpaired *t*-test, Fisher's exact test, one-way analysis of variance (ANOVA) or two-way repeated measures ANOVA with Tukey's *post hoc* were used for statistical comparison between groups, as indicated.

## Additional information

**How to cite this article:** Ghosh, D. *et al*. VAMP7 regulates constitutive membrane incorporation of the cold-activated channel TRPM8. *Nat. Commun.* 7:10489 doi: 10.1038/ncomms10489 (2016).

## Supplementary Material

Supplementary Information Supplementary Figures 1-10 and Supplementary Table 1

Supplementary Movie 1 TIRF movie showing the movement of LAMP1-GFP and TRPM8-mCherry in HEK293 cells.

Supplementary Movie 2 TIRF movie showing the movement of TRPM4-GFP and TRPM8-mCherry in HEK293 cells.

Supplementary Movie 3 TIRF movie showing the movement of LAMP1-GFP and TRPM8-mCherry in HEK293 cells following photobleaching of the evanescent field.

Supplementary Movie 4 TIRF movie showing the movement of VAMP7-GFP and TRPM8-mCherry in HEK293 cells following photobleaching of the evanescent field.

Supplementary Movie 5 TIRF movie showing the movement of VAMP7-GFP and TRPM8-mCherry in F11 cells.

Supplementary Movie 6 TIRF movie showing the movement of TRPM8-GFP and TRPM8-mCherry in HEK293 cells.

## References

[b1] Vriens J., Nilius B. & Voets T. Peripheral thermosensation in mammals. Nat. Rev. Neurosci. 15, 573–589 (2014). 25053448 10.1038/nrn3784

[b2] Bautista D. M. . The menthol receptor TRPM8 is the principal detector of environmental cold. Nature 448, 204–208 (2007). 17538622 10.1038/nature05910

[b3] Colburn R. W. . Attenuated cold sensitivity in TRPM8 null mice. Neuron 54, 379–386 (2007). 17481392 10.1016/j.neuron.2007.04.017

[b4] Dhaka A. . TRPM8 is required for cold sensation in mice. Neuron 54, 371–378 (2007). 17481391 10.1016/j.neuron.2007.02.024

[b5] McKemy D. D., Neuhausser W. M. & Julius D. Identification of a cold receptor reveals a general role for TRP channels in thermosensation. Nature 416, 52–58 (2002). 11882888 10.1038/nature719

[b6] Peier A. M. . A TRP channel that senses cold stimuli and menthol. Cell 108, 705–715 (2002). 11893340 10.1016/s0092-8674(02)00652-9

[b7] Almeida M. C. . Pharmacological blockade of the cold receptor TRPM8 attenuates autonomic and behavioural cold defenses and decreases deep body temperature. J. Neurosci. 32, 2086–2099 (2012). 22323721 10.1523/JNEUROSCI.5606-11.2012 PMC3566779

[b8] Knowlton W. M., Daniels R. L., Palkar R., McCoy D. D. & McKemy D. D. Pharmacological blockade of TRPM8 ion channels alters cold and cold pain responses in mice. PLoS ONE 6, e25894 (2011). 21984952 10.1371/journal.pone.0025894 PMC3184174

[b9] Xing H., Chen M., Ling J., Tan W. & Gu J. G. TRPM8 mechanism of cold allodynia after chronic nerve injury. J. Neurosci. 27, 13680–13690 (2007). 18077679 10.1523/JNEUROSCI.2203-07.2007 PMC6673615

[b10] Proudfoot C. J. . Analgesia mediated by the TRPM8 cold receptor in chronic neuropathic pain. Curr. Biol. 16, 1591–1605 (2006). 16920620 10.1016/j.cub.2006.07.061

[b11] Bandell M. . High-throughput random mutagenesis screen reveals TRPM8 residues specifically required for activation by menthol. Nat. Neurosci. 9, 493–500 (2006). 16520735 10.1038/nn1665

[b12] Janssens A. & Voets T. Ligand stoichiometry of the cold- and menthol-activated channel TRPM8. J. Physiol. 589, 4827–4835 (2011). 21878524 10.1113/jphysiol.2011.216523 PMC3224877

[b13] Voets T. . The principle of temperature-dependent gating in cold- and heat-sensitive TRP channels. Nature 430, 748–754 (2004). 15306801 10.1038/nature02732

[b14] Voets T., Owsianik G., Janssens A., Talavera K. & Nilius B. TRPM8 voltage sensor mutants reveal a mechanism for integrating thermal and chemical stimuli. Nat. Chem. Biol. 3, 174–182 (2007). 17293875 10.1038/nchembio862

[b15] Zakharian E., Cao C. & Rohacs T. Gating of transient receptor potential melastatin 8 (TRPM8) channels activated by cold and chemical agonists in planar lipid bilayers. J. Neurosci. 30, 12526–12534 (2010). 20844147 10.1523/JNEUROSCI.3189-10.2010 PMC2989179

[b16] Liu B. & Qin F. Functional control of cold- and menthol-sensitive TRPM8 ion channels by phosphatidylinositol 4,5-bisphosphate. J. Neurosci. 25, 1674–1681 (2005). 15716403 10.1523/JNEUROSCI.3632-04.2005 PMC6725927

[b17] Rohacs T., Lopes C. M., Michailidis I. & Logothetis D. E. PI(4,5)P2 regulates the activation and desensitization of TRPM8 channels through the TRP domain. Nat. Neurosci. 8, 626–634 (2005). 15852009 10.1038/nn1451

[b18] Zhang X. . Direct inhibition of the cold-activated TRPM8 ion channel by Galphaq. Nat. Cell Biol. 14, 851–858 (2012). 22750945 10.1038/ncb2529 PMC3428855

[b19] Camprubi-Robles M., Planells-Cases R. & Ferrer-Montiel A. Differential contribution of SNARE-dependent exocytosis to inflammatory potentiation of TRPV1 in nociceptors. FASEB J. 23, 3722–3733 (2009). 19584302 10.1096/fj.09-134346

[b20] Stein A. T., Ufret-Vincenty C. A., Hua L., Santana L. F. & Gordon S. E. Phosphoinositide 3-kinase binds to TRPV1 and mediates NGF-stimulated TRPV1 trafficking to the plasma membrane. J. Gen. Physiol. 128, 509–522 (2006). 17074976 10.1085/jgp.200609576 PMC2151588

[b21] Zhang X., Huang J. & McNaughton P. A. NGF rapidly increases membrane expression of TRPV1 heat-gated ion channels. EMBO J. 24, 4211–4223 (2005). 16319926 10.1038/sj.emboj.7600893 PMC1356334

[b22] Schmidt M., Dubin A. E., Petrus M. J., Earley T. J. & Patapoutian A. Nociceptive signals induce trafficking of TRPA1 to the plasma membrane. Neuron 64, 498–509 (2009). 19945392 10.1016/j.neuron.2009.09.030 PMC2854037

[b23] Toro C. A. . Agonist-dependent modulation of cell surface expression of the cold receptor TRPM8. J. Neurosci. 35, 571–582 (2015). 25589752 10.1523/JNEUROSCI.3820-13.2015 PMC6605369

[b24] Jahn R. & Fasshauer D. Molecular machines governing exocytosis of synaptic vesicles. Nature 490, 201–207 (2012). 23060190 10.1038/nature11320 PMC4461657

[b25] Burgo A. . A molecular network for the transport of the TI-VAMP/VAMP7 vesicles from cell centre to periphery. Dev. Cell 23, 166–180 (2012). 22705394 10.1016/j.devcel.2012.04.019

[b26] Hesketh G. G. . VARP is recruited on to endosomes by direct interaction with retromer, where together they function in export to the cell surface. Dev. Cell 29, 591–606 (2014). 24856514 10.1016/j.devcel.2014.04.010 PMC4059916

[b27] Proux-Gillardeaux V., Raposo G., Irinopoulou T. & Galli T. Expression of the Longin domain of TI-VAMP impairs lysosomal secretion and epithelial cell migration. Biol. Cell 99, 261–271 (2007). 17288539 10.1042/BC20060097

[b28] Danglot L. . Absence of TI-VAMP/Vamp7 leads to increased anxiety in mice. J. Neurosci. 32, 1962–1968 (2012). 22323709 10.1523/JNEUROSCI.4436-11.2012 PMC6621696

[b29] Veliz L. A. . Near-membrane dynamics and capture of TRPM8 channels within transient confinement domains. PLoS ONE 5, e13290 (2010). 20948964 10.1371/journal.pone.0013290 PMC2952625

[b30] Vercauteren D. . Dynamic colocalization microscopy to characterize intracellular trafficking of nanomedicines. ACS Nano 5, 7874–7884 (2011). 21923168 10.1021/nn2020858

[b31] Saftig P. & Klumperman J. Lysosome biogenesis and lysosomal membrane proteins: trafficking meets function. Nat. Rev. Mol. Cell Biol. 10, 623–635 (2009). 19672277 10.1038/nrm2745

[b32] Mindell J. A. Lysosomal acidification mechanisms. Annu. Rev. Physiol. 74, 69–86 (2012). 22335796 10.1146/annurev-physiol-012110-142317

[b33] Ghosh D., Segal A. & Voets T. Distinct modes of perimembrane TRP channel turnover revealed by TIR-FRAP. Sci. Rep. 4, 7111 (2014). 25407951 10.1038/srep07111 PMC4236744

[b34] Melli G. & Hoke A. Dorsal root ganglia sensory neuronal cultures: a tool for drug discovery for peripheral neuropathies. Exp. Opin. Drug Discov. 4, 1035–1045 (2009). 10.1517/17460440903266829 PMC2908326 20657751

[b35] Rao S. K., Huynh C., Proux-Gillardeaux V., Galli T. & Andrews N. W. Identification of SNAREs involved in synaptotagmin VII-regulated lysosomal exocytosis. J. Biol. Chem. 279, 20471–20479 (2004). 14993220 10.1074/jbc.M400798200

[b36] Schoch S. . SNARE function analysed in synaptobrevin/VAMP knockout mice. Science 294, 1117–1122 (2001). 11691998 10.1126/science.1064335

[b37] Ryan T. A. Kiss-and-run fuse-pinch-and-linger, fuse-and-collapse: the life and times of a neurosecretory granule. Proc. Natl Acad. Sci. USA 100, 2171–2173 (2003). 12606723 10.1073/pnas.0530260100 PMC151312

[b38] Vanweert A. W. M., Dunn K. W., Geuze H. J., Maxfield F. R. & Stoorvogel W. Transport from late endosomes to lysosomes, but not sorting of integral membrane-proteins in endosomes, depends on the vacuolar proton pump. J. Cell Biol. 130, 821–834 (1995). 7642700 10.1083/jcb.130.4.821 PMC2199957

[b39] Martinez-Arca S., Alberts P., Zahraoui A., Louvard D. & Galli T. Role of tetanus neurotoxin insensitive vesicle-associated membrane protein (TI-VAMP) in vesicular transport mediating neurite outgrowth. J. Cell Biol. 149, 889–900 (2000). 10811829 10.1083/jcb.149.4.889 PMC2174569

[b40] Vivona S. . The longin SNARE VAMP7/TI-VAMP adopts a closed conformation. J. Biol. Chem. 285, 17965–17973 (2010). 20378544 10.1074/jbc.M110.120972 PMC2878558

[b41] Vandewauw I., Owsianik G. & Voets T. Systematic and quantitative mRNA expression analysis of TRP channel genes at the single trigeminal and dorsal root ganglion level in mouse. BMC. Neurosci. 14, 21 (2013). 23410158 10.1186/1471-2202-14-21 PMC3576292

[b42] Karashima Y. . Bimodal action of menthol on the transient receptor potential channel TRPA1. J. Neurosci. 27, 9874–9884 (2007). 17855602 10.1523/JNEUROSCI.2221-07.2007 PMC6672629

[b43] Hjerling-Leffler J., Alqatari M., Ernfors P. & Koltzenburg M. Emergence of functional sensory subtypes as defined by transient receptor potential channel expression. J. Neurosci. 27, 2435–2443 (2007). 17344381 10.1523/JNEUROSCI.5614-06.2007 PMC6672507

[b44] Karashima Y. . TRPA1 acts as a cold sensor *in vitro* and *in vivo*. Proc. Natl Acad. Sci. USA 106, 1273–1278 (2009). 19144922 10.1073/pnas.0808487106 PMC2633575

[b45] Gentry C., Stoakley N., Andersson D. A. & Bevan S. The roles of iPLA2, TRPM8 and TRPA1 in chemically induced cold hypersensitivity. Mol. Pain 6, 4 (2010). 20092626 10.1186/1744-8069-6-4 PMC2822744

[b46] Andrejewski N. . Normal lysosomal morphology and function in LAMP-1-deficient mice. J. Biol. Chem. 274, 12692–12701 (1999). 10212251 10.1074/jbc.274.18.12692

[b47] Nishino I. . Primary LAMP-2 deficiency causes X-linked vacuolar cardiomyopathy and myopathy (Danon disease). Nature 406, 906–910 (2000). 10972294 10.1038/35022604

[b48] Eskelinen E. L. . Disturbed cholesterol traffic but normal proteolytic function in LAMP-1/LAMP-2 double-deficient fibroblasts. Mol. Biol. Cell 15, 3132–3145 (2004). 15121881 10.1091/mbc.E04-02-0103 PMC452571

[b49] Huynh K. K. . LAMP proteins are required for fusion of lysosomes with phagosomes. EMBO J. 26, 313–324 (2007). 17245426 10.1038/sj.emboj.7601511 PMC1783450

[b50] Lippincott-Schwartz J. & Fambrough D. M. Cycling of the integral membrane glycoprotein, LEP100, between plasma membrane and lysosomes: kinetic and morphological analysis. Cell 49, 669–677 (1987). 3107839 10.1016/0092-8674(87)90543-5

[b51] Eskelinen E. L., Tanaka Y. & Saftig P. At the acidic edge: emerging functions for lysosomal membrane proteins. Trends Cell Biol. 13, 137–145 (2003). 12628346 10.1016/s0962-8924(03)00005-9

[b52] Cheng X. . The intracellular Ca(2)(+) channel MCOLN1 is required for sarcolemma repair to prevent muscular dystrophy. Nat. Med. 20, 1187–1192 (2014). 25216637 10.1038/nm.3611 PMC4192061

[b53] Brauchi S., Orio P. & Latorre R. Clues to understanding cold sensation: thermodynamics and electrophysiological analysis of the cold receptor TRPM8. Proc. Natl Acad. Sci. USA 101, 15494–15499 (2004). 15492228 10.1073/pnas.0406773101 PMC523461

[b54] Coco S. . Subcellular localization of tetanus neurotoxin-insensitive vesicle-associated membrane protein (VAMP)/VAMP7 in neuronal cells: evidence for a novel membrane compartment. J. Neurosci. 19, 9803–9812 (1999). 10559389 10.1523/JNEUROSCI.19-22-09803.1999 PMC6782963

[b55] Hua Z. . v-SNARE composition distinguishes synaptic vesicle pools. Neuron 71, 474–487 (2011). 21835344 10.1016/j.neuron.2011.06.010 PMC3155686

[b56] Vriens J. . TRPM3 is a nociceptor channel involved in the detection of noxious heat. Neuron 70, 482–494 (2011). 21555074 10.1016/j.neuron.2011.02.051

[b57] Kirchhausen T. Clathrin. Annu. Rev. Biochem. 69, 699–727 (2000). 10966473 10.1146/annurev.biochem.69.1.699

[b58] Nabi I. R. & Le P. U. Caveolae/raft-dependent endocytosis. J. Cell Biol. 161, 673–677 (2003). 12771123 10.1083/jcb.200302028 PMC2199359

[b59] Mu F. T. . EEA1, an early endosome-associated protein. EEA1 is a conserved alpha-helical peripheral membrane protein flanked by cysteine "fingers" and contains a calmodulin-binding IQ motif. J. Biol. Chem. 270, 13503–13511 (1995). 7768953 10.1074/jbc.270.22.13503

[b60] Gorvel J. P., Chavrier P., Zerial M. & Gruenberg J. rab5 controls early endosome fusion *in vitro*. Cell 64, 915–925 (1991). 1900457 10.1016/0092-8674(91)90316-q

[b61] Van Der Sluijs P. . The small GTP-binding protein rab4 is associated with early endosomes. Proc. Natl Acad. Sci. USA 88, 6313–6317 (1991). 1906178 10.1073/pnas.88.14.6313 PMC52073

[b62] Takahashi S. . Rab11 regulates exocytosis of recycling vesicles at the plasma membrane. J. Cell Sci. 125, 4049–4057 (2012). 22685325 10.1242/jcs.102913

[b63] Bucci C., Thomsen P., Nicoziani P., McCarthy J. & van Deurs B. Rab7: a key to lysosome biogenesis. Mol. Biol. Cell 11, 467–480 (2000). 10679007 10.1091/mbc.11.2.467 PMC14786

[b64] Vanoevelen J. . The secretory pathway Ca2+/Mn2+-ATPase 2 is a Golgi-localized pump with high affinity for Ca2+ ions. J. Biol. Chem. 280, 22800–22808 (2005). 15831496 10.1074/jbc.M501026200

[b65] Winther A. M. . The sarcolipin-bound calcium pump stabilizes calcium sites exposed to the cytoplasm. Nature 495, 265–269 (2013). 23455424 10.1038/nature11900

[b66] Bergmann J. E. Using temperature-sensitive mutants of vsv to study membrane-protein biogenesis. Methods Cell Biol. 32, 85–110 (1989). 2558277 10.1016/s0091-679x(08)61168-1

[b67] Grynkiewicz G., Poenie M. & Tsien R. Y. A new generation of Ca2+ indicators with greatly improved fluorescence properties. J. Biol. Chem. 260, 3440–3450 (1985). 3838314

