# Supplementary material for: VAMP7 regulates constitutive membrane incorporation of the cold-activated channel TRPM8
Source: Nat Commun. 2016 Feb 4;7:10489. doi: 10.1038/ncomms10489 (PMC4742910; doi:10.1038/ncomms10489)
Supplement: Supplementary Information — Supplementary Figures 1-10 and Supplementary Table 1 [file ncomms10489-s1.pdf]

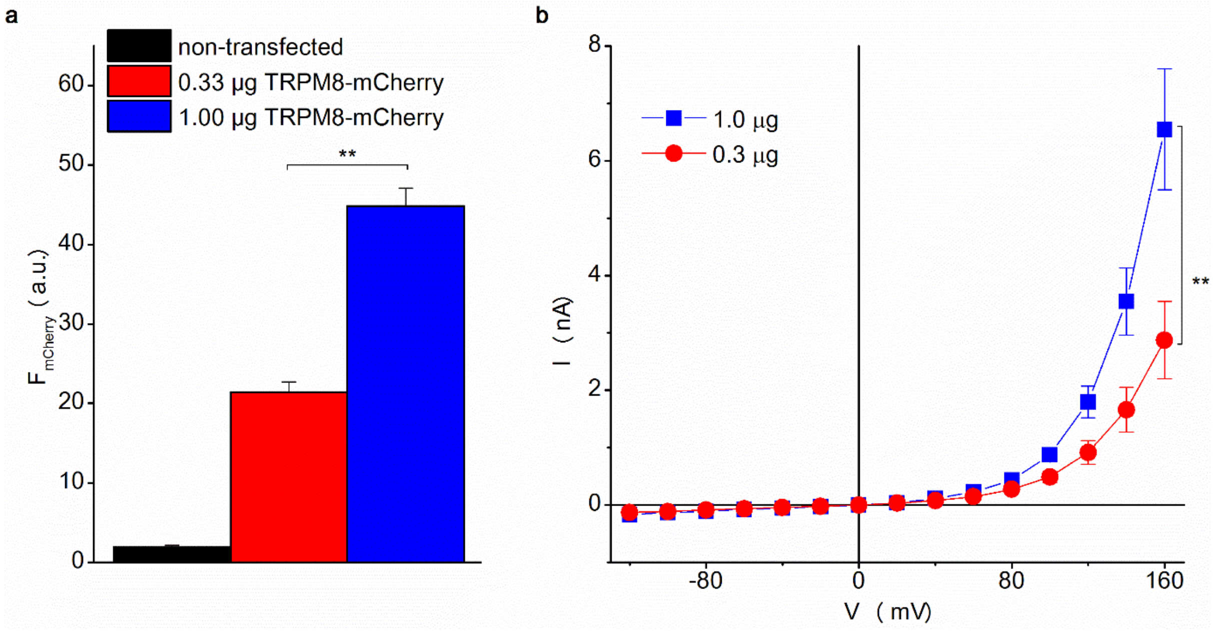

Supplementary Figure 1

(a) Average mCherry fluorescence in HEK293 cells transfected with 0.33 or 1 µg of TRPM8-mCherry plasmid.

(b) Average TRPM8 current in HEK293 cells transfected with 0.33 or 1 µg of TRPM8-mCherry plasmid.

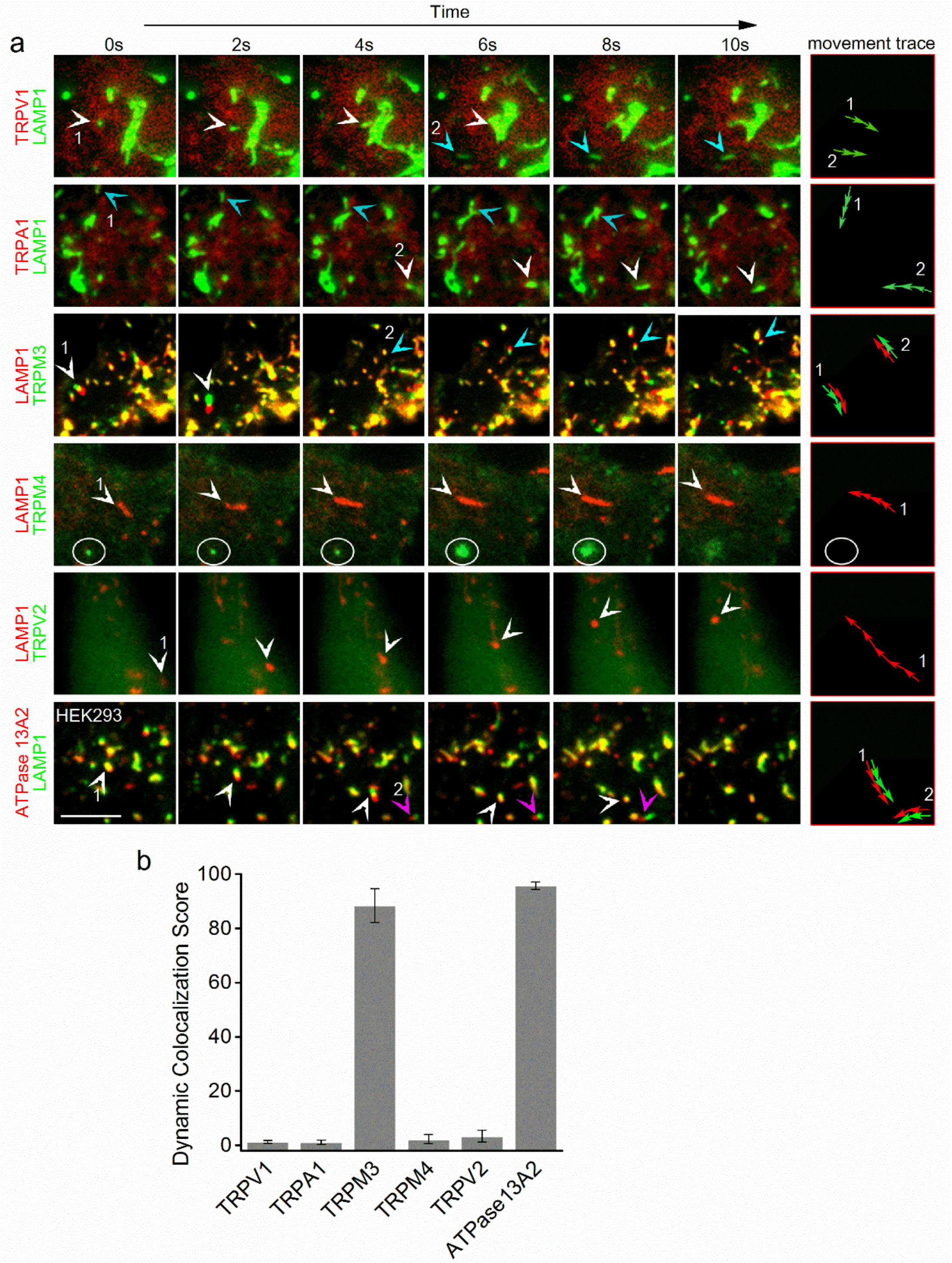

Supplementary Figure 2

(a) Dual-color TIRF images at consecutive intervals showing the movement of the indicated mCherry-coupled (in red) along with the indicated GFP-coupled marker proteins (in green) following co-expression in HEK293 cells. The arrow heads point at moving structures that were tracked in consecutive images. The last column traces the movement of the structures marked by arrowheads in corresponding rows. In the fourth row, the circle marks a TRPM4-positive vesicle that fuses with the plasma membrane. n > 8 cells for each condition. Scale bar, 5 µm.

(b) Quantification of the dynamic colocalization of the indicated TRP channels and ATPase13A2 with LAMP1 (n=8-12). ATPase13A2 is used as a positive control for colocalization with LAMP1, based on literature{Holemans, 2015 #69}.

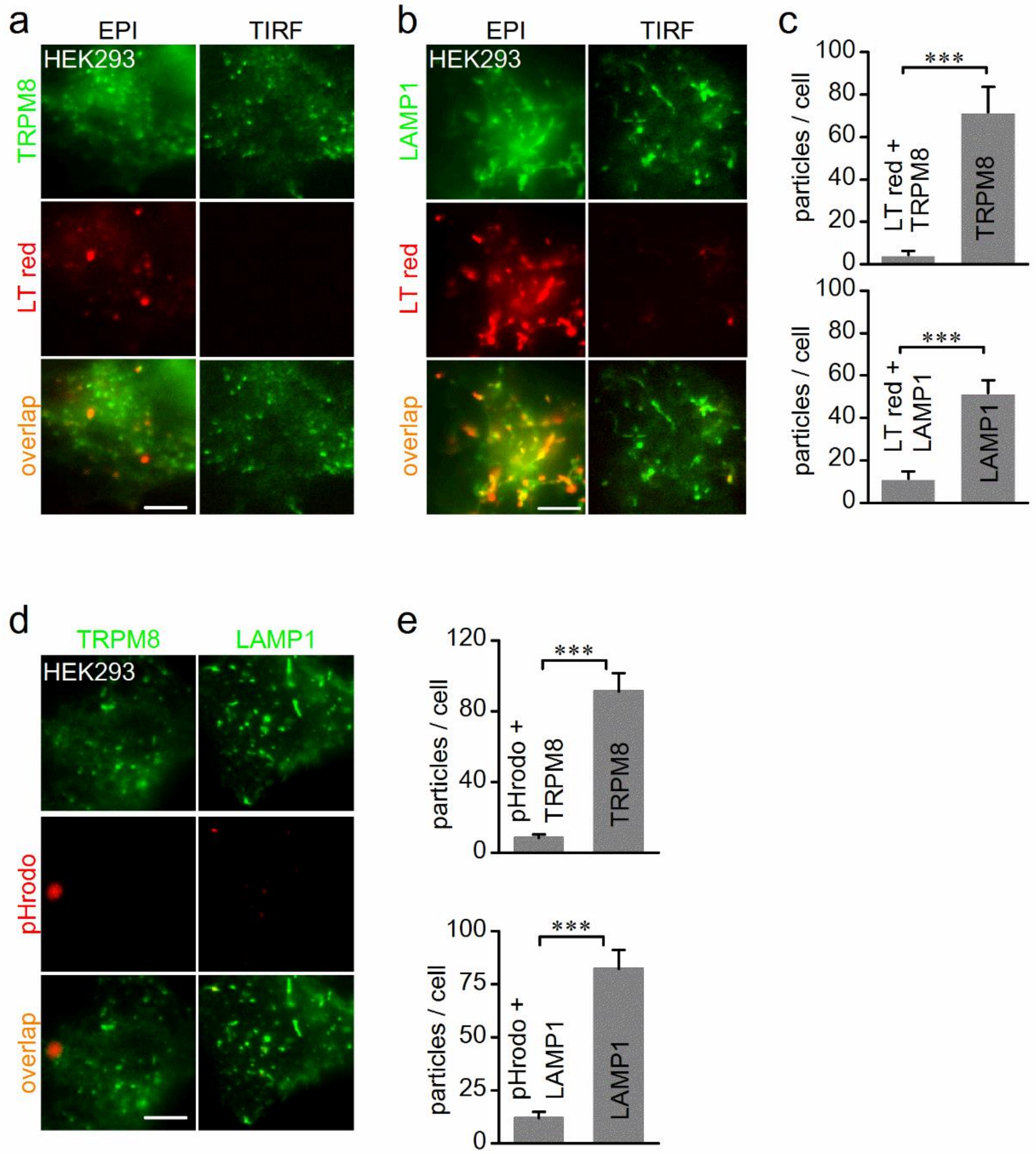

Supplementary Figure 3

(a, b) Epifluorescence (EPI) and TIRF images illustrating TRPM8-GFP (**a**) and LAMP1-GFP (**b**) fluorescence in HEK293 cells treated with 50nM of Lysotracker red (LT red) for 30 minutes. Scale bar, 5 µm.

(c) Quantification of the number of near-membrane TRPM8-GFP (n=10 cells) or LAMP1-GFP (n=10 cells) structures that are positive for LT red, as observed in TIRF mode. Significance is determined by Two Sample Independent t-Test. *** $P < 0.001$

(d) TIRF images illustrating TRPM8-GFP and LAMP1-GFP fluorescence in HEK293 cells treated with treated with pHrodo red dextran at 20µg/ml for 30 minutes. Scale bar, 5 µm.

(e) Quantification of the number of near-membrane TRPM8-GFP (n=28 cells) or LAMP1-GFP (n=24 cells) structures that are positive for pHrodo red dextran, as observed in TIRF mode. Significance is determined by Two Sample Independent t-Test. *** $P < 0.001$.

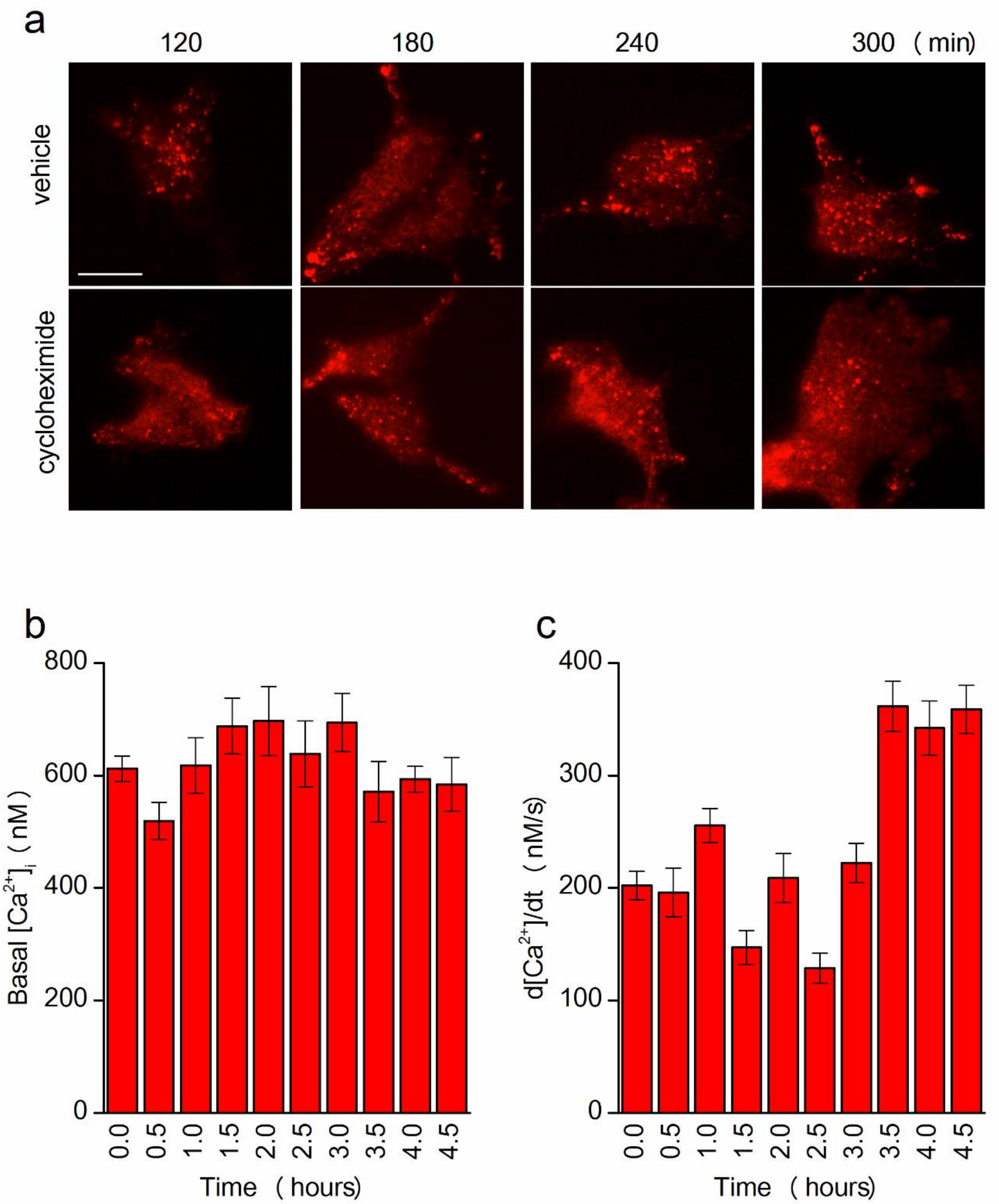

Supplementary Figure 4

a) TIRF images of HEK293 cells transfected with TRPM8-mCherry treated for the indicated time periods with cycloheximide (100µM) or vehicle. Scale bar, 10µm.

(b-c) Resting $[Ca^{2+}]_i$ and rate of $[Ca^{2+}]_i$ rise in response to menthol on TRPM8-mCherry-transfected HEK293 cells treated for the indicated time periods with cycloheximide. Data are shown as mean ± SEM. (n > 60 cells for each condition).

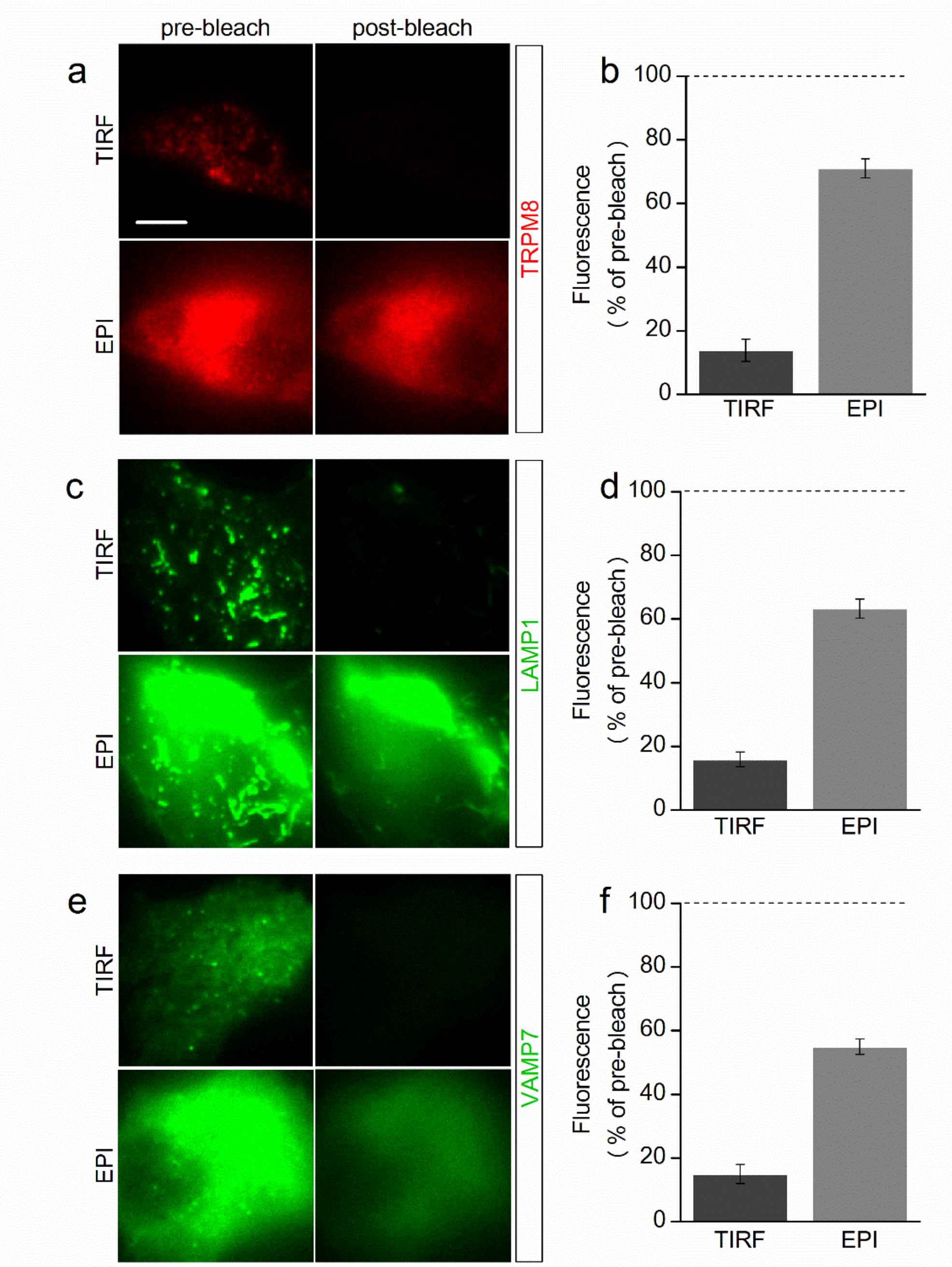

Supplementary Figure 5

(a,c,e) Images obtained using TIRF and EPI-fluorescence before and after the high-intensity TIR illumination step in HEK293 cells transfected with TRPM8, LAMP1 and VAMP7. Scale bar, 5µm.

(b,d,f) Post-bleach fluorescence measured in TIR and epi mode, normalized to the respective pre-bleach fluorescence levels.

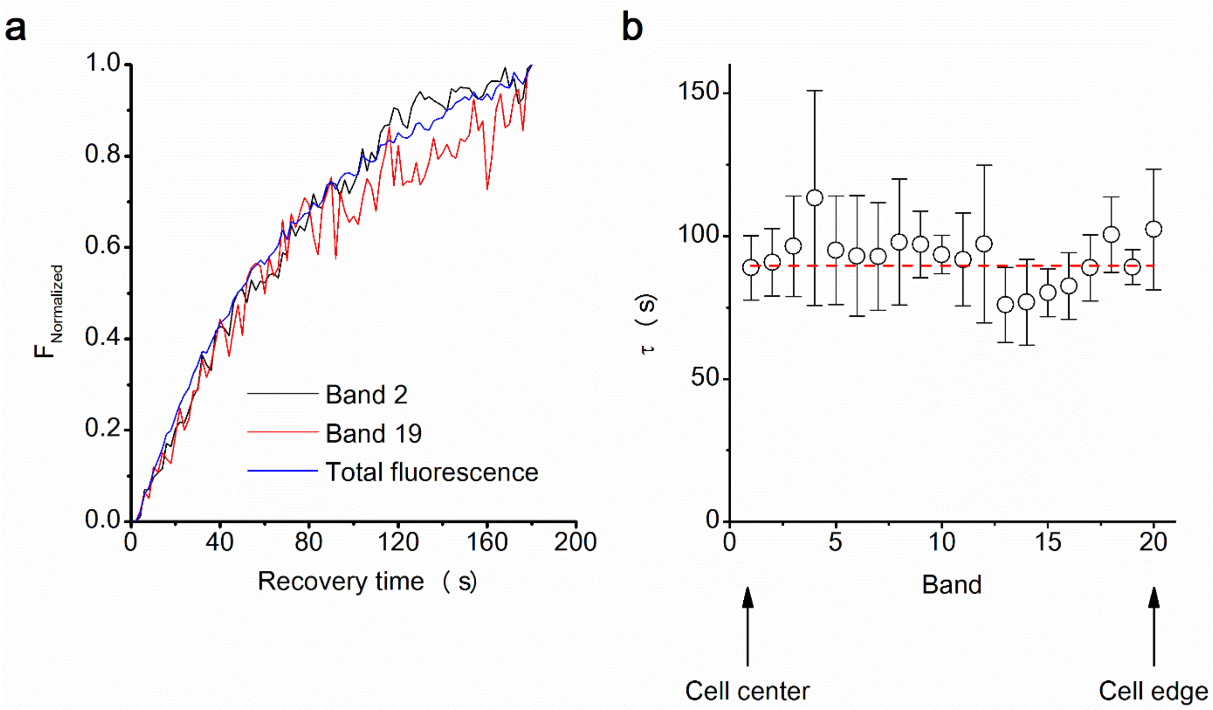

Supplementary Figure 6

- **(a)** To analyze spatio-temporal aspects of the recovery of VAMP7-pHluorin fluorescence in experiments like as in **Fig. 6a**, the cell footprint was subdivided in 20 equally shaped and equidistant bands as described in ref. 43. Shown is a representative example showing the normalized total fluorescence of the entire footprint, along with a band at the cell center (band 19) and at the cell periphery (band 2).
- **(b)** Exponential time constants for the recovery of fluorescence in the different bands. The constant time course in the different bands indicates that fluorescence recovery mainly occurs from inside the cell towards the plasma membrane rather than through lateral diffusion from non-bleached areas of the plasma membrane.

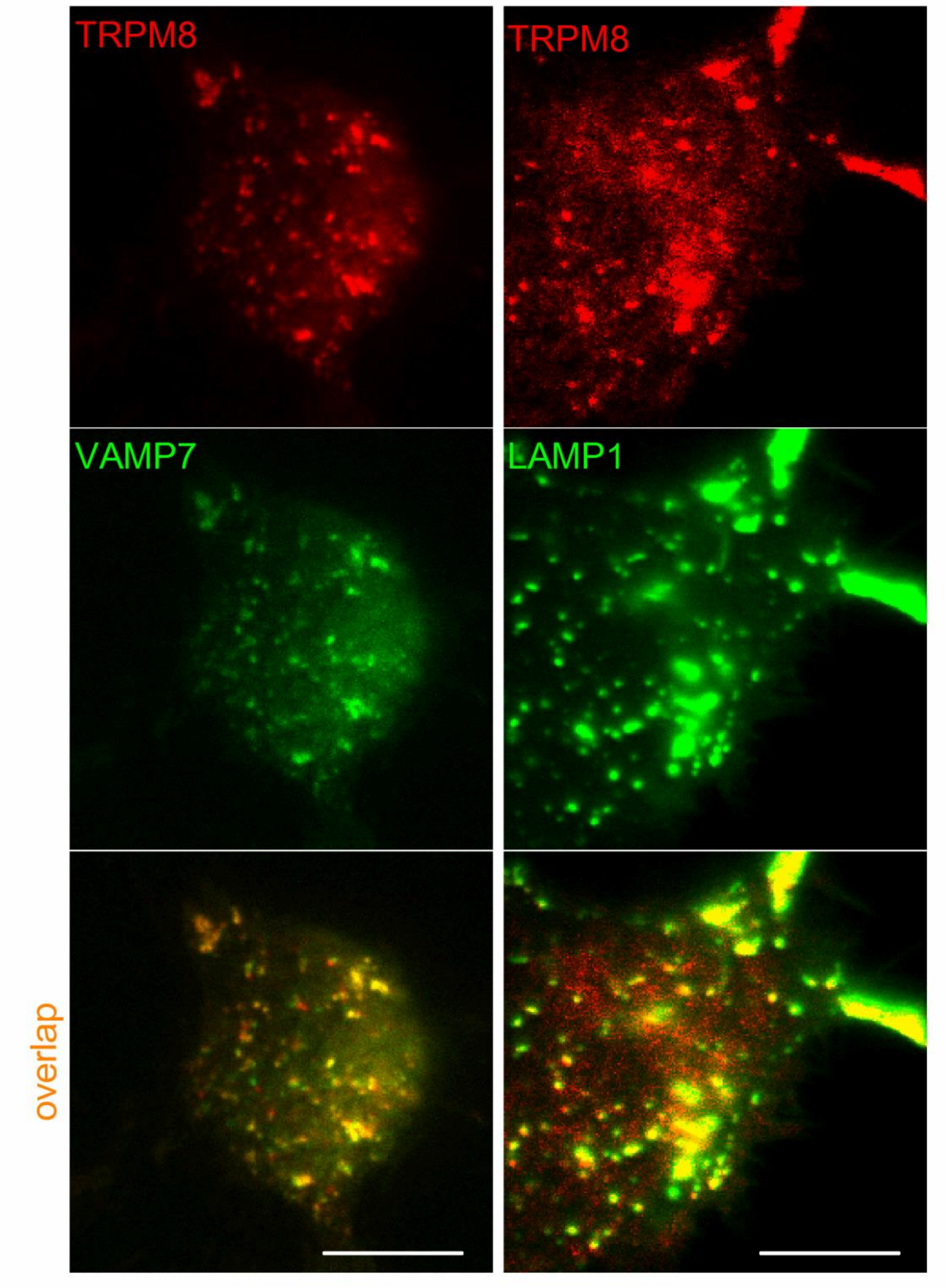

Supplementary Figure 7

Dual-color TIRF images showing colocalization of TRPM8 and VAMP7 (left) and TRPM8 and LAMP1 (right) in F11 cells. Scale bar, 10μm.

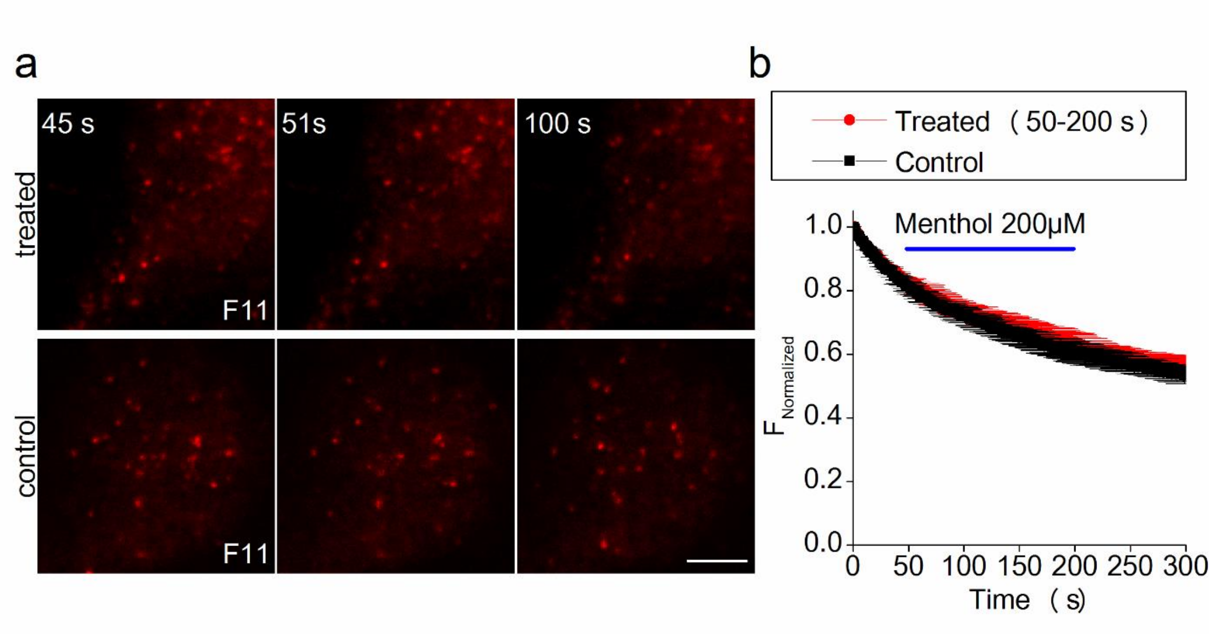

Supplementary Figure 8

TIRF images (**a**) and corresponding total fluorescence (**b**) of TRPM8-mCherry in F11 cells, showing the lack of effect of menthol (200 μM) on total TRPM8 fluorescence intensity. Scale bar, 5μm.

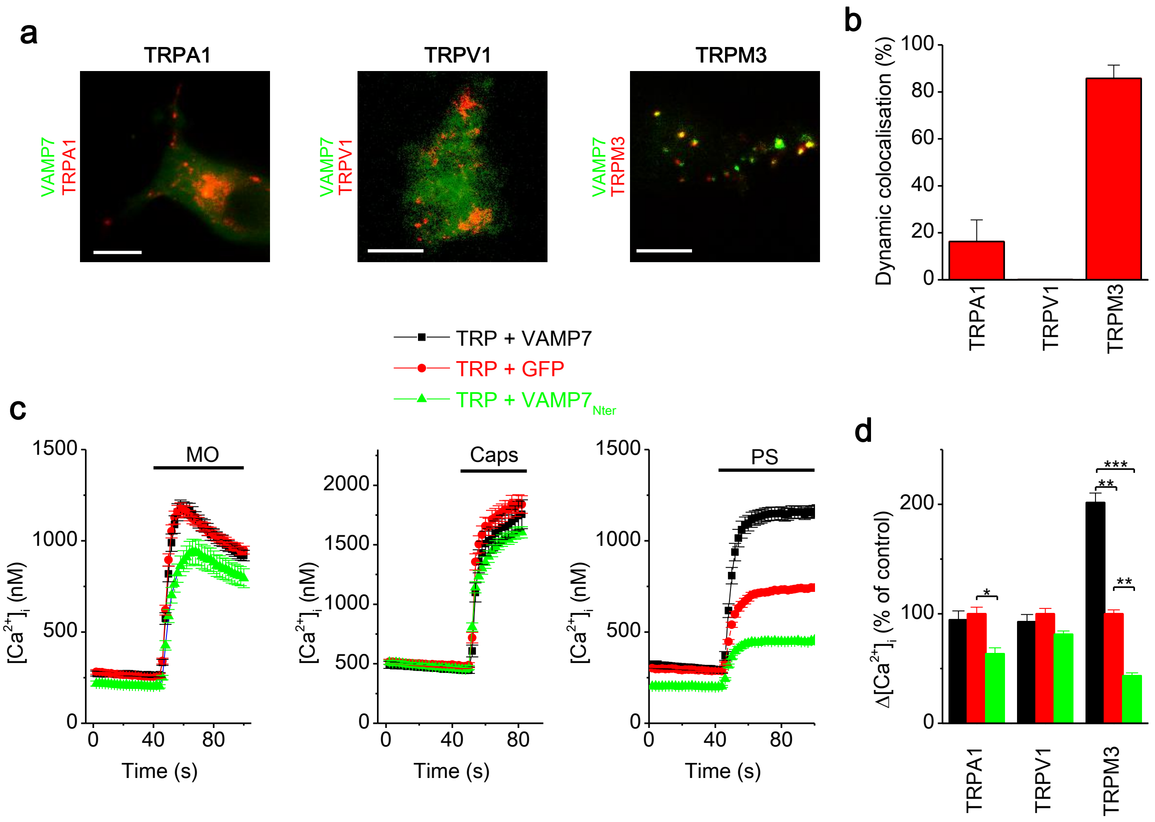

Supplementary Figure 9

- **(a)** Dual-color TIRF images showing VAMP7-GFP together with the indicated mCherry-tagged TRP channels. Scale bar, 10µm
- **(b)** Quantification of the dynamic colocalization of VAMP7 and the indicated TRP channels.
- **(c)** Time course of $[Ca^{2+}]_i$ in HEK293 cells expressing TRPA1 (left), TRPV1 (middle) or TRPM3 (right) along with either VAMP7, GFP, or $VAMP7_{Nter}$. Horizontal bars indicate application of the respective agonists mustard oil (MO; 50 µM), capsaicin (caps; 1 µM) or pregnenolone sulphate (PS; 50 µM).
- **(d)** Effect of WT VAMP7 and $VAMP7_{Nter}$ on the agonist-induced responses of the three TRP channels, normalized to the response in control (GFP-expressing) cells. Significance was determined by 1-Way ANOVA with Tukey's post-hoc test; *, $P < 0.05$;**, $P < 0.01$.

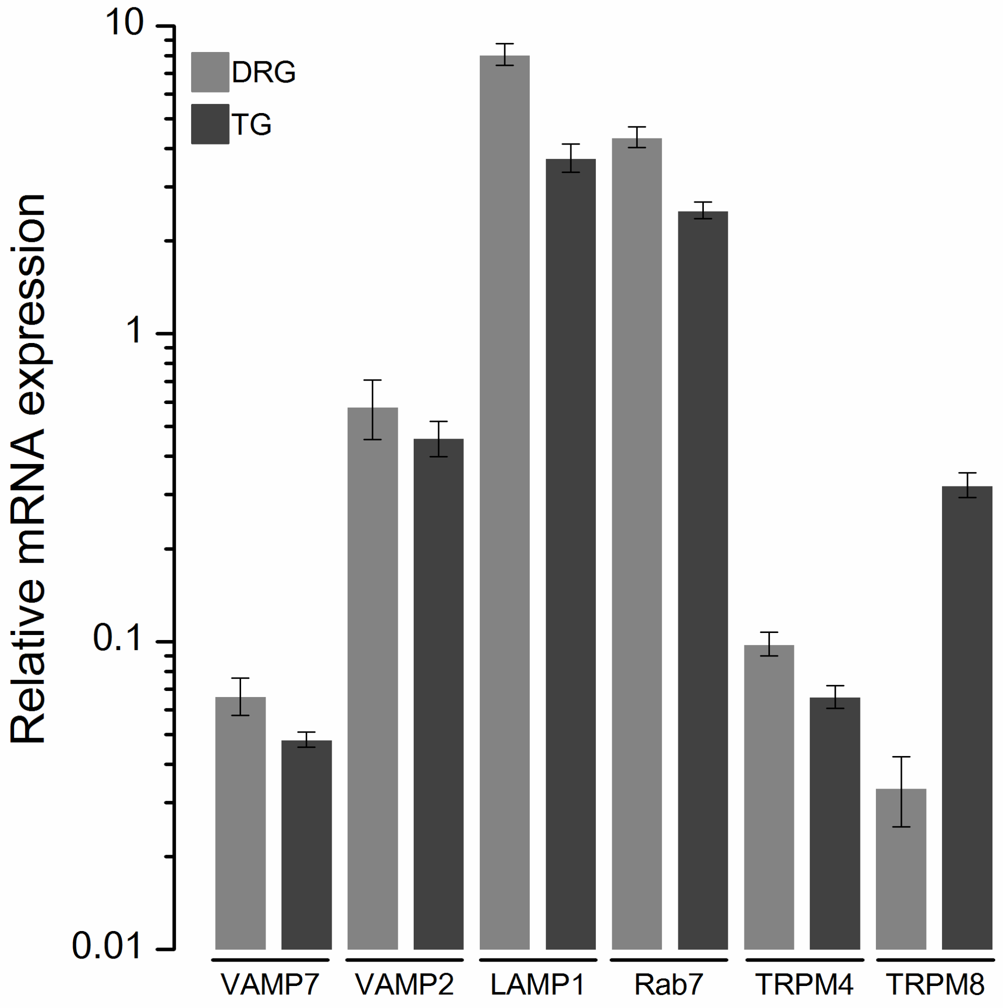

Supplementary Figure 10

Quantitative RT-PCR performed on TG and DRG from WT C57BL/6N female mice. Level of mRNA expression was normalized to PGK1 mRNA expression. (n = 4, independent experiments). PCR-primers are described in **Supplementary Table 1**.

Supplementary Table 1: Q-PCR assays

Gene name	Assay ID	Reference Sequence	Exon boundary	Assay location	Amplicon length
VAMP7	Mm00807071_m1	NM_011515.4	5 -6	571	144
LAMP1	Mm01217069_m1	NM_010684.2	5-6	981	79
RAB7	Mm01183732_g1	NM_009005.2	4- 5	524	77
VAMP2	Mm01325243_m1	NM_009497.3	4- 5	425	117
TRPM8	Mm00454566_m1	NM_134252.3	22 - 23	3116	89
TRPM4	Mm00613173_m1	NM_175130.4	9 - 10	1225	78
PGK1	Mm00435617_m1	NM_008828.2	5 - 6	651	137
HPRT1	Mm00446968_m1	NM_013556.2	6 - 7	630	65
